# Pinnipeds and avian influenza: a global timeline and review of research on the impact of highly pathogenic avian influenza on pinniped populations with particular reference to the endangered Caspian seal (*Pusa caspica*)

**DOI:** 10.3389/fcimb.2024.1325977

**Published:** 2024-06-26

**Authors:** Alimurad Gadzhiev, Guy Petherbridge, Kirill Sharshov, Ivan Sobolev, Alexander Alekseev, Marina Gulyaeva, Kirill Litvinov, Ivan Boltunov, Abdulgamid Teymurov, Alexander Zhigalin, Madina Daudova, Alexander Shestopalov

**Affiliations:** ^1^ Institute of Ecology and Sustainable Development, Dagestan State University, Makhachkala, Russia; ^2^ Caspian Centre for Nature Conservation, International Institute of Ecology and Sustainable Development, Association of Universities and Research Centers of Caspian Region States, Makhachkala, Russia; ^3^ Research Institute of Virology, Federal Research Centre for Fundamental and Translational Medicine, Siberian Branch, Russian Academy of Sciences, Novosibirsk, Russia; ^4^ Department of Natural Sciences, Novosibirsk State University, Novosibirsk, Russia; ^5^ Laboratory of Ecological and Biological Research, Astrakhan State Nature Biosphere Reserve, Astrakhan, Russia; ^6^ Department of Vertebrate Zoology, Faculty of Biology, M.V. Lomonosov Moscow State University, Moscow, Russia

**Keywords:** pinnipeds, Caspian seal, avian influenza viruses, HPAI H5N1, Caspian Sea nature conservation, marine mammals, surveillance, phylogenetics

## Abstract

This study reviews chronologically the international scientific and health management literature and resources relating to impacts of highly pathogenic avian influenza (HPAI) viruses on pinnipeds in order to reinforce strategies for the conservation of the endangered Caspian seal (*Pusa caspica*), currently under threat from the HPAI H5N1 subtype transmitted from infected avifauna which share its haul-out habitats. Many cases of mass pinniped deaths globally have occurred from HPAI spill-overs, and are attributed to infected sympatric aquatic avifauna. As the seasonal migrations of Caspian seals provide occasions for contact with viruses from infected migratory aquatic birds in many locations around the Caspian Sea, this poses a great challenge to seal conservation. These are thus critical locations for the surveillance of highly pathogenic influenza A viruses, whose future reassortments may present a pandemic threat to humans.

## Introduction

This study surveys the extensive international scientific literature and health management initiatives relating to impacts of highly pathogenic avian influenza A HPAI) viruses on pinnipeds in order to reinforce strategies for the conservation of the endangered Caspian seal (*Pusa caspica*), under threat from the HPAI H5N1 subtype transmitted from infected avifauna which share its haul-out habitats. It is intended to facilitate the understanding of the widespread recent deaths in pinnipeds associated with the spread of highly pathogenic avian influenza A viruses by providing a detailed global timeline of outbreaks together with their location, the clinical pathologies of dead or stranded pinnipeds and virological analyses of samples taken from them. With an extensive current bibliography, it is intended primarily as a working guide or “one stop bird flu information base” for those engaged in the rescue and conservation of pinnipeds, be this from the practical point of view of a field worker or from the perspective of an environmental manager or policy maker, with particular emphasis placed on recent and potential impacts of viral zoonoses on the Caspian seal and associated bird life, both major indicators of the state of the Caspian Sea’s littoral and marine ecosystem.

## The Caspian sea region: historical depopulation of Caspian seals

**Figure 1:**
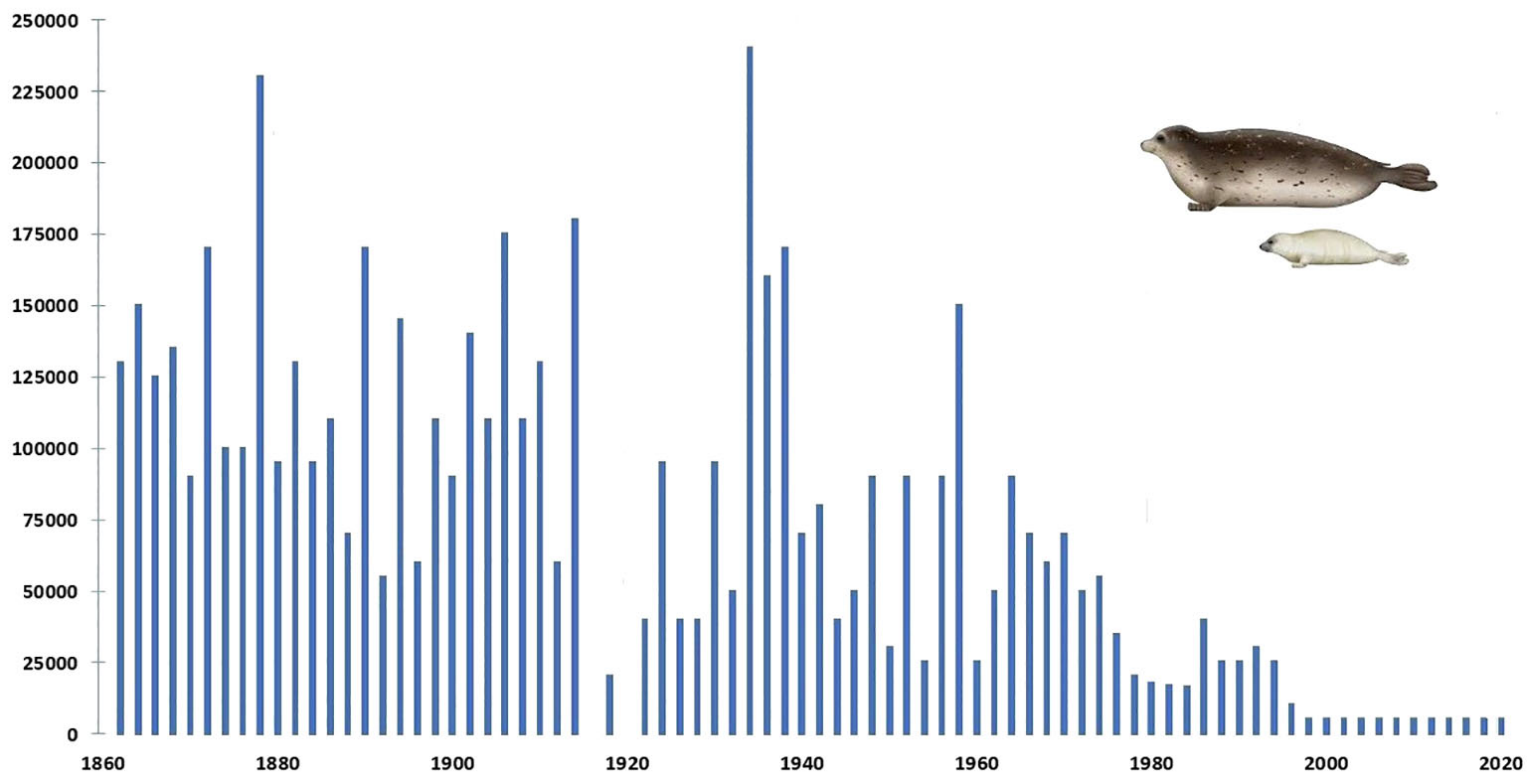
Graph plotting annual numbers of Caspian seals harvested between 1867 and 2020 (none were culled after 2000). Compiled from: FAO Fishsta+; Caspian Environment Programme, Transboundary Analysis Revisit (2007); and Härkönen et al. (2012). The latter assessed significantly less seals born in most of these years than were harvested.

The population of the Caspian seal (*Pusa caspica*) declined from an estimated one million at the end of the 19^th^ century to some 50,000 in the first decade of the 21^st^ century, primarily due to state sanctioned “harvesting” (Dorofeev and Freiman, 1928; Roganov, 1932; Badamshin, 1969), (**Figure 1**) but also for a range of other reasons which have increasingly impacted on its viability. The dynamics of many of these are as yet not adequately understood. However, almost all impacts have some anthropogenic element: ecological disturbance, climate-change related, epidemiological, overfishing, entanglement in marine litter and nets, habitat degradation, marine pollutants, disruption of natural food resources, including due to invasion of non-endemic species. The first recorded mass mortality of Caspian seals occurred on the Tyuleniy (Seal) islands of Dagestan, Russia in 1931 (Timoshenko, 1969; Kydyrmanov et al., 2023). Further mass seal deaths of varying proportions were recorded in 1955-56, 1997, 2000, 2007, 2020 and 2022.

Following particularly large mass mortality events along the northern and central Caspian Sea coast in 2022 (numbered as many as 10,000) local fisherman and specialists in the conservation of this iconic pinniped have observed a very substantial decrease in its numbers in Russian waters. Studies internationally have observed a correlation between deaths in aquatic birds due to various strains of Highly Pathogenic Avian Influenza Viruses (HPAIV) and mass deaths of seals in shared habitats also due to HPAIV. Mass and individual deaths in aquatic birds due to the HPAI H5N1 subtype have recently been recorded in habitats also frequented by the Caspian seal (as reported in detail below).

## Character and etiology of avian influenza viruses and their transmission to pinnipeds

Avian influenza viruses (AIVs) are type A influenza viruses of avifauna from which all influenza A viruses affecting other animals are thought to have derived, regardless of their host species (EU, 2006; Taubenberger, 2008; Van Tam and Sellwood, 2010; DeLiberto et al., 2011; OIE, 2015). AIVs are widespread and in circulation amongst many wild bird species, in particular aquatic birds of the Anseriformes (mainly ducks, geese and swans) and Charadriiformes (mainly pelagic birds, gulls, terns [Laridae] and waders) orders, which constitute their principal natural host reservoirs (Easterday et al., 2006; Stallknecht and Brown, 2009; Curran et al., 2013; Sobolev et al., 2016; Gulyaeva et al., 2021).

Avian influenza viruses (AIVs) usually result in very mild gastrointestinal tract infections in wild avifauna, which result in low and temporary immunity, with co-circulation of and co-infection with multiple other strains and subtypes. As infected birds usually remain asymptomatic, AIVs may circulate undetected. During a brief period of infection (c. one month) the virus replicates in the gut and is then excreted in the feces at very high levels. Wild avifauna can congregate in large numbers before prior or during migration, thus providing optimal conditions for the transmission and mixing of AIVs and thus maintaining the reservoir in certain species (Van Tam and Sellwood, 2010).

Those AIVs which lead to mild or no disease and may circulate without being detected are considered as low pathogenic avian influenza viruses (LPAIVs) (Van Tam and Sellwood, 2010). However, LPAIV subtypes H5 and H7 may evolve towards highly pathogenic avian influenza viruses (HPAIVs) when transmitted into farmed poultry populations. Thus, the HPAIV H5N1 subtype which emerged in South-East China in 1997 and has since continued to circulate in poultry, has led to hundreds of cases of severe respiratory infection in humans and systemic disease in a wide range of wild, farmed and domestic avian and mammalian species (Chen et al., 2017; Hill et al., 2018; Sharshov et al., 2019; Cui et al., 2022) (see further below).

The adherence of migratory aquatic avifauna to habitual transcontinental and intercontinental flyways has resulted in AIVs being phylogenetically separated into 2 genetically distinct lineages, Eurasian (EA) and North American (NA) (Webster et al., 1992). In zones where flyways overlap, AIVs with gene segments of both NA and EA lineages have been isolated, although these instances are rare. The monitoring of wild bird viruses in western (Alaska) and eastern (Newfoundland) North America has identified a significant majority of AIVs which have whole-genome segments from EA lineage viruses but only very infrequently have reassortant AIVs s with both EA and NA gene segment constellations having been isolated from them (Huang et al., 2014; Ramey et al., 2015). In 2014, for example, the H5N8 virus, a reassortant of goose/Guangdong/1/96 (Gs/GD) H5Nx lineage was identified in wild aquatic birds along the North American sector of the Pacific Americas littoral flyway. NA lineage AIVs reasserted with this particular subtype, leading to severe losses in the Canadian and United States poultry industry (Berhane et al., 2022). See also Vahlenkamp and Harder (2006), Ip et al. (2008), Wille et al. (2011a, b) and Lee et al. (2015).

**Figure 2:**
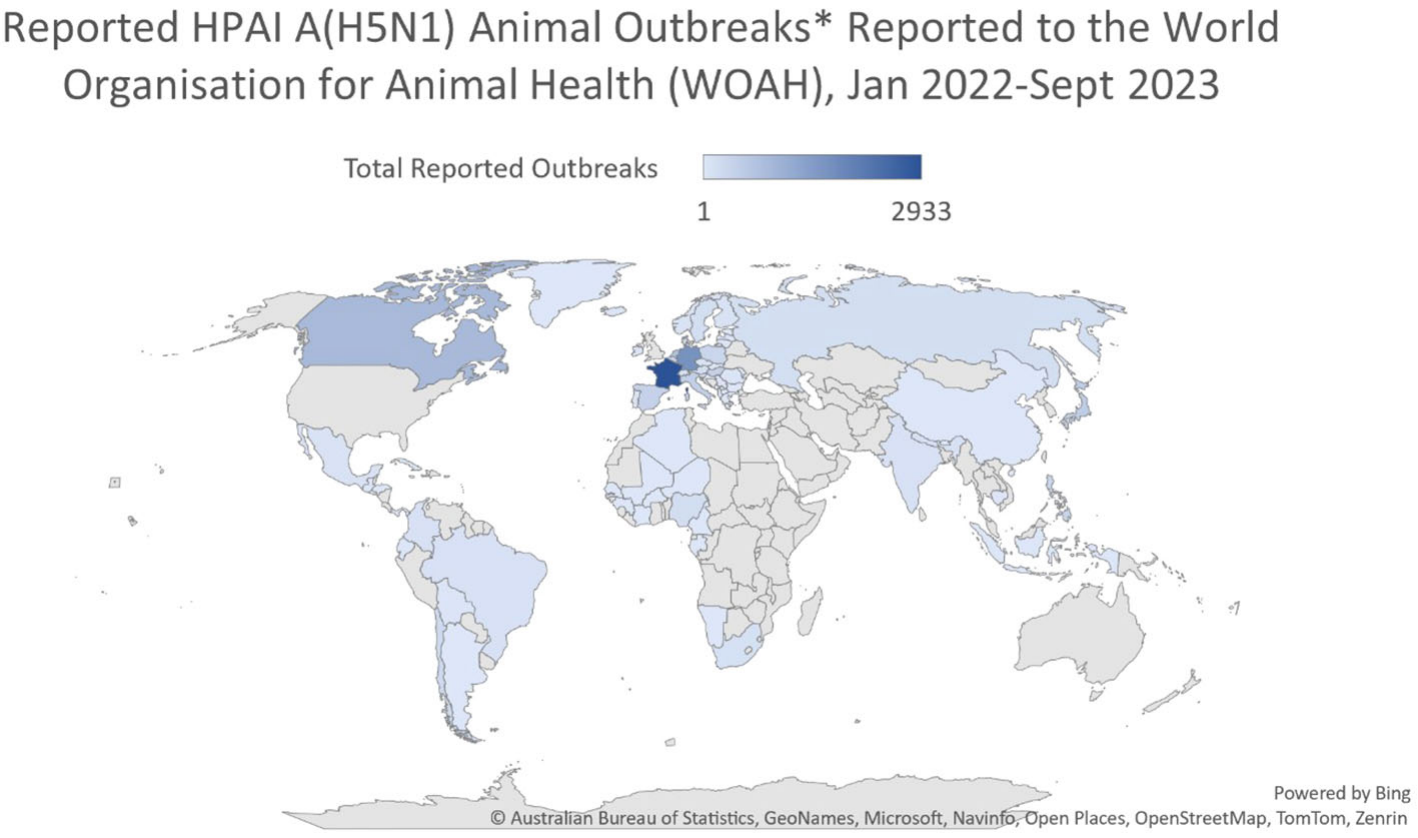
Reported HPAI A(H5N1) animal outbreaks reported to the World Organization for Animal Health (WOAH), January 2022-June 2023. After: Australian Bureau of Statistics, Geonames, Microsoft, Navinfo, Open Street Map, Tom Tom, Zenrin.

**Figure 3:**
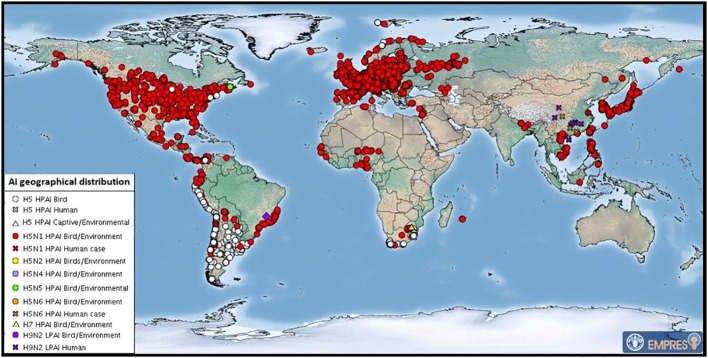
July 2023 map of global distribution of AIV with zoonotic potential (including H5Nx HPAI viruses) since 1 October 2022. Food and Agricultural Organization of the United Nations. These are outbreaks in animals officially reported since the previous update (22 HPAI subtypes: H5 (untyped) (53), H5N1 (625), H5N5 (2), H5N6 (1) and H7 (4). After FAO: https://www.fao.org/animal-health/situation-updates/global-aiv-with-zoonotic-potential/en.

AIVs originating in wild avifauna can breach the host species barrier and infect certain terrestrial mammals as sporadic cases of infection, self-limiting epidemics, or sustained epidemics that may eventually develop into recurring epidemics through reassortment. After crossing the species barrier, some AIVs which cross the species interface can become established in new hosts and circulate independently of their reservoir hosts (Hatta et al., 2001; Berhane et al., 2022). Since 2021, there has been a significant change in the eco-geography of the highly pathogenic avian influenza A subtype H5N1 (particularly clade 2.3.4.4b), which is currently circulating on five continents (Kilpatrick et al., 2006; Lloren et al., 2017; Isoda et al., 2022) (**Figures 2**, **3**).

The planet’s air and water together are serving as an interconnected environmental continuum within which certain avian and mammalian taxa (including marine mammals) are serving both as a contact source and a sink for avian influenza viruses which are being transmitted uni-directionally, contra-directionally (bi-directionally), and in multiple directions as they seasonally migrate and also move long-habitual locations in response to climate change or in search of accessible food sources (Breban et al., 2009; Tian et al., 2014). The HPAI H5N1 virus is reaching places previously not known to have been exposed to such a virulent AIV strain and has the potential to spread into endangered species with major impacts on biodiversity.

Various species of marine mammals have been shown to be vulnerable to AIV infection, particularly pinnipeds, which are exposed at haul-out and breeding sites and interface ecologically with wild birds and with other infected sympatric mammalian species (Venkatesh et al., 2020). As laid out in the global timeline of seal infections below, previous outbreaks and individually reported infections have shown that seals may be affected by and die as a result of infection with H10N7, H3N8, H7N7, H4N6, and other AIVs (Venkatesh et al., 2020). As indicated above, AIVs in birds replicate principally in the gastrointestinal tract intestine and is transmitted orally or via feces and other bodily secretions (respiratory tropism and oropharyngeal shedding have also been observed) (Webster et al., 1978; Daoust et al., 2011; Daoust et al., 2013; França et al., 2012; Höfle et al., 2012). Avian influenza viruses can survive in the natural environment for quite long periods (Stallknecht et al., 1990; Brown et al., 2009) in conditions which permit exchange of viruses between pinnipeds and aquatic avifauna, which share habitats and nutritional sources. See also Harvell et al., 1999; Horm et al., 2012).

A number of instances of marine mammal infection caused by many AIV subtypes, including H1N1, H3N3, H3N8, H4N5, H4N6, H7N7, and H10N7, have been substantiated with effects ranging from mass mortalities to sub-clinical; in a majority of these infections avian influenza was determined as the source (Webster et al., 1981a; Danner et al., 1998; Ohishi, 2002; Ramis et al., 2012; White, 2013; Karlsson et al., 2014; Fereidouni et al., 2014; Berhane et al., 2022). There are also studies which indicate that the grey seal (*Halichoerius grypus*) may constitute an endemic viral reservoir from which influenza A viruses can be transmitted in coastal environments to other mammals and aquatic avifauna and may potentially also infect humans (Duignan et al., 1995; Duignan et al., 1997; Herfst et al., 2012; Puryear et al., 2016; Dittrich et al., 2018). In this regard, levels of seroprevalence in healthy grey seal populations (20–26%) (Bodewes et al., 2015a, b; Puryear et al., 2016) have been found to be comparable with those of wild bird populations (31–60%) depending on other factors such as species, geography, season, etc. (Fereidouni et al., 2010; Wilson et al., 2013; Curran et al., 2015; Venkatesh et al., 2020). Nevertheless, as White (2013) suggests, it seems that severe AIV infection is sporadic in pinnipeds and does not usually result in mass mortalities. Further investigation is required to explore the causes, which are likely to be multifactorial of such epizootics and the evolution of influenza viruses and associated mortality events in pinnipeds (White, 2013; Baz et al., 2015; Belser et al., 2011).

## Recent significant Caspian seal and wild avifauna mortality events in the Caspian sea region 2022-2023

The Caspian seal (*Pusa caspica*) is the only marine mammal inhabiting the Caspian Sea (Eybatov, 2010; Food and Agriculture Organization of the United Nations (FAO), 2023). As the top predator in the marine food chain, the state of its population is a key indicator of the integrity of the region’s biodiversity. It has been classified as endangered in the Red List of Threatened Species of the IUCN since 2008.

Between 31 March and 2 May 2022, over 832 dead Caspian seals were found along the Caspian Sea coast of the Mangystau region of Kazakhstan. Carcasses found by Kazakhstan’s Institute of Hydrobiology and Ecology were in varying states of decomposition and may have died at different times and of varying causes, including external signs of pneumonia (possibly symptomatic of an avian influenza infection). In such a situation, it was impossible to take useful histological, virological and other samples. Later in the year, in November 2022, the Fishery Committee of the Kazakhstan Ministry of Ecology, Geology and Natural Resources reported their representatives had found 141 dead Caspian seals on the Kazakhstan coast between Bautino and Fort-Shevchenko on the Tyub-Karagan peninsula. Specialists from scientific organizations, including the Norwegian Institute of Bioeconomy Research (NIBIO), and Kazakhtstan government entities, concluded that the principal cause of death for most of the seals was virus-associated acute pneumonia as a result of an outbreak of mixed influenza and morbillivirus infection (https://partner.sciencenorway.no/climate-endangered-species-environment/norwegian-kazakhstan-research-collaboration-aims-to-save-endangered-seal-species/2164060 Accessed 09.10.2023).

Since that mass mortality event, another was recorded in Kazakhstan coastal areas between March and early June 2023 numbering 343 deal seals (including 73 pups) (http://kaspika.org/ru/2023/06/09/hundreds-of-dead-caspian-seals-in-kazakhstan-1/Accessed 09.10.2023). A determination of the cause or causes has not yet been published.

In early December 2022, thousands of dead seals in varying advanced states of decomposition were also found along the coast of Dagestan, Russia, mainly between Izberbash and Sulak. Specialists from the Institute of Ecology and Sustainable Development (DSU) and representatives of the Dagestan Ministry of Natural Resources and Ecology examined more than 100 dead seals and took samples of pathological material from six of the least decomposed carcasses for toxicological and virological analyses (five of which proved unsuitable for laboratory analysis, as reflected in the results given below). External examination of the animals showed that most had a characteristic discharge from the nasal and oral cavities in the form of bloody secretions, which, according to studies referenced below, are typical when animals die from asphyxiation as a result of respiratory tract infection when infected with avian influenza.

Subsequently, samples taken for virological analyses on 10 December 2022, were sent to the Research Institute of Virology (FRCFTM SB RAS) in Novosibirsk for testing for the presence of avian influenza and the morbillivirus CVD. An internal report of the Research Institute of Virology dated 31 January 2023 states that: (1) Laboratory analysis using PCR with primers for morbilliviruses, determined that all samples gave a negative result; that is RNA of morbilliviruses (including canine distemper and seal distemper) was not detected; and (b) in analysis using PCR with primers for influenza type A virus, samples from five animals were negative, while samples from one seal (internal organs and nasal swabs) were positive for influenza type A virus.

However, due to the fact that all the corpses of animals discovered, including those from which samples were taken, had died more than a day before the collection of pathological material, inhibiting isolation of influenza A virus RNA and specification of the subtype, it was concluded that further investigation was required, optimally through testing for antibodies in surviving seals in the same Russian waters.

**Figure 4:**
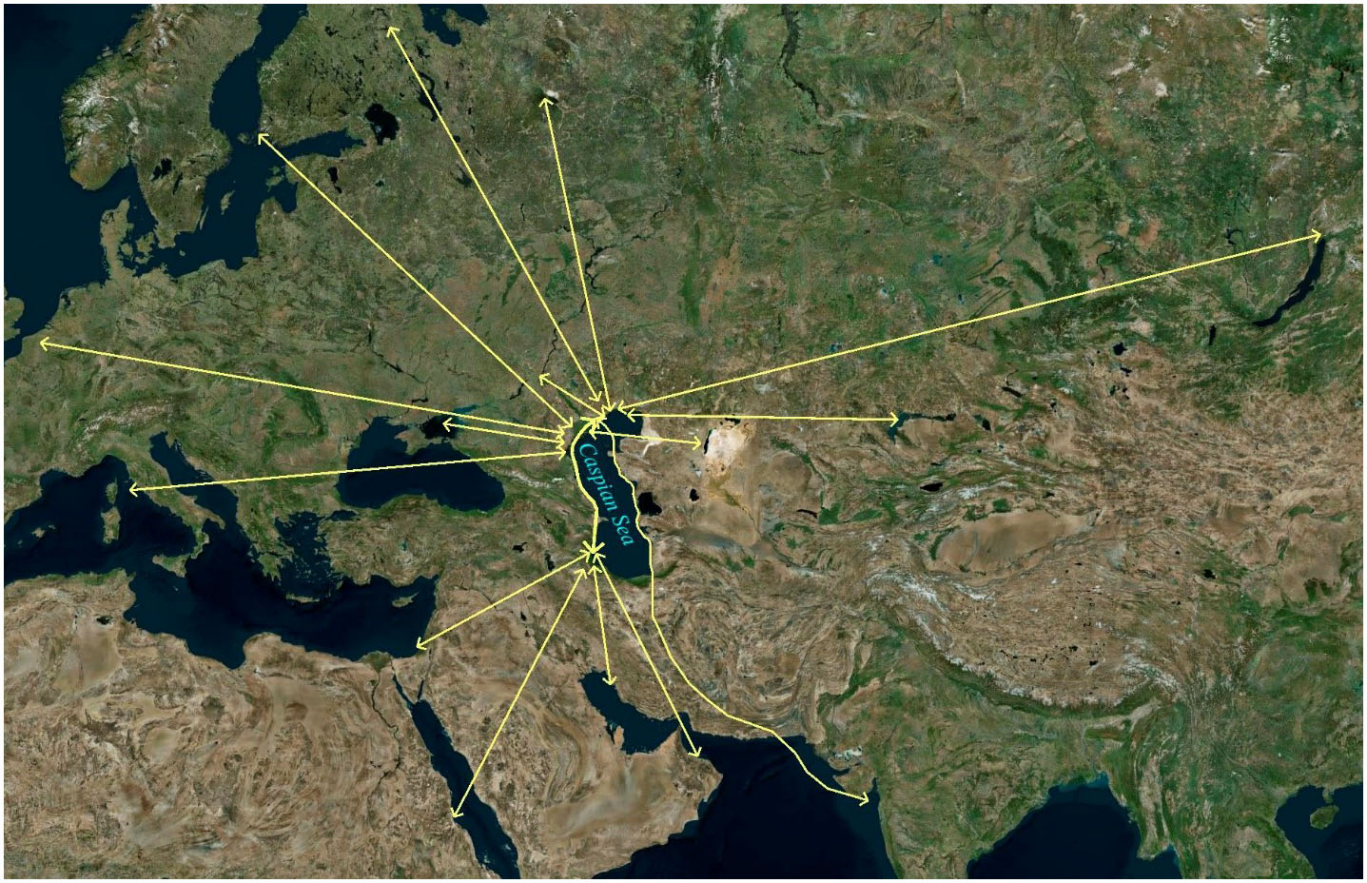
Map showing the hub of a number of migratory flyways used by aquatic birds in the northern area of the Caspian Sea where Maliy Zhemchuzhniy Island is located. Derived from: Google Earth & Vilkov, 2016b. https://doi.org/10.1134/S199542551603015X.

**Figure 5:**
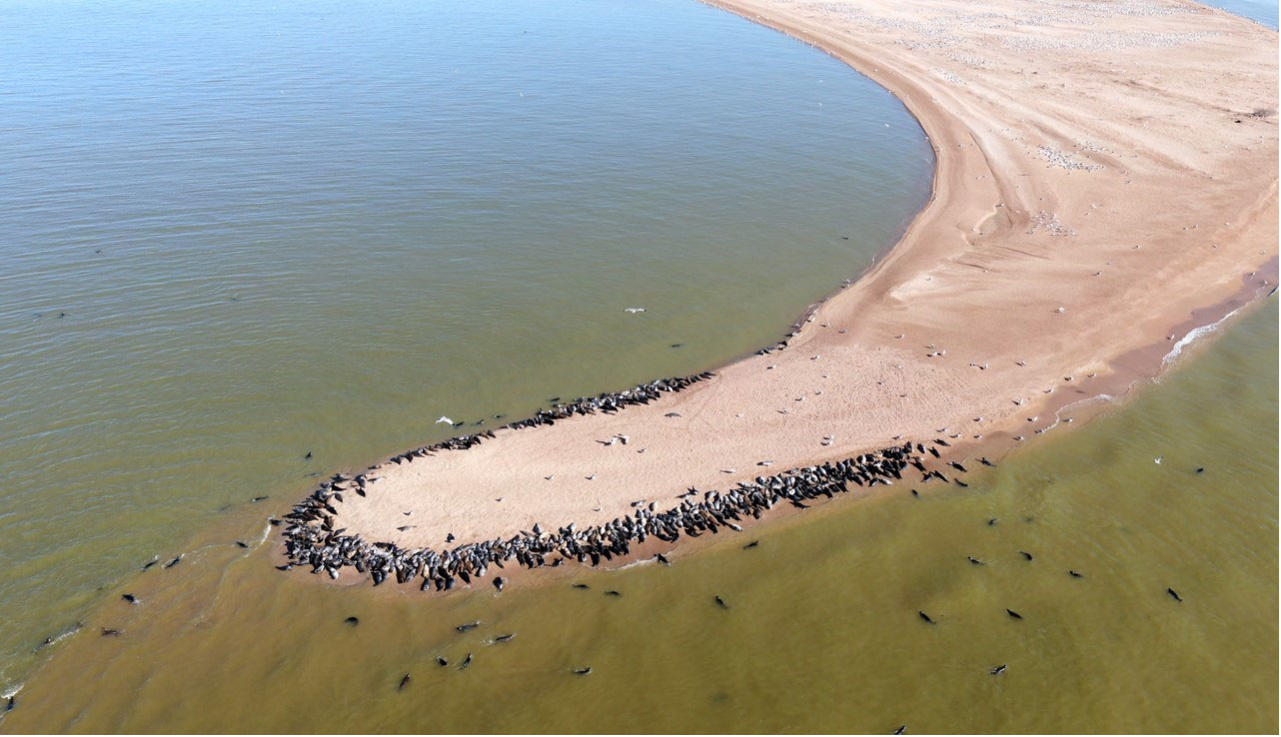
Southern tip of Maliy Zhemchuzhniy Island, northern Caspian Sea, Russia, showing Caspian seals in proximity to aquatic birds and their breeding colonies (great black-headed gulls, Caspian gulls and Caspian terns). Photo: ASNBR, 11 April 2020.

**Figure 6:**
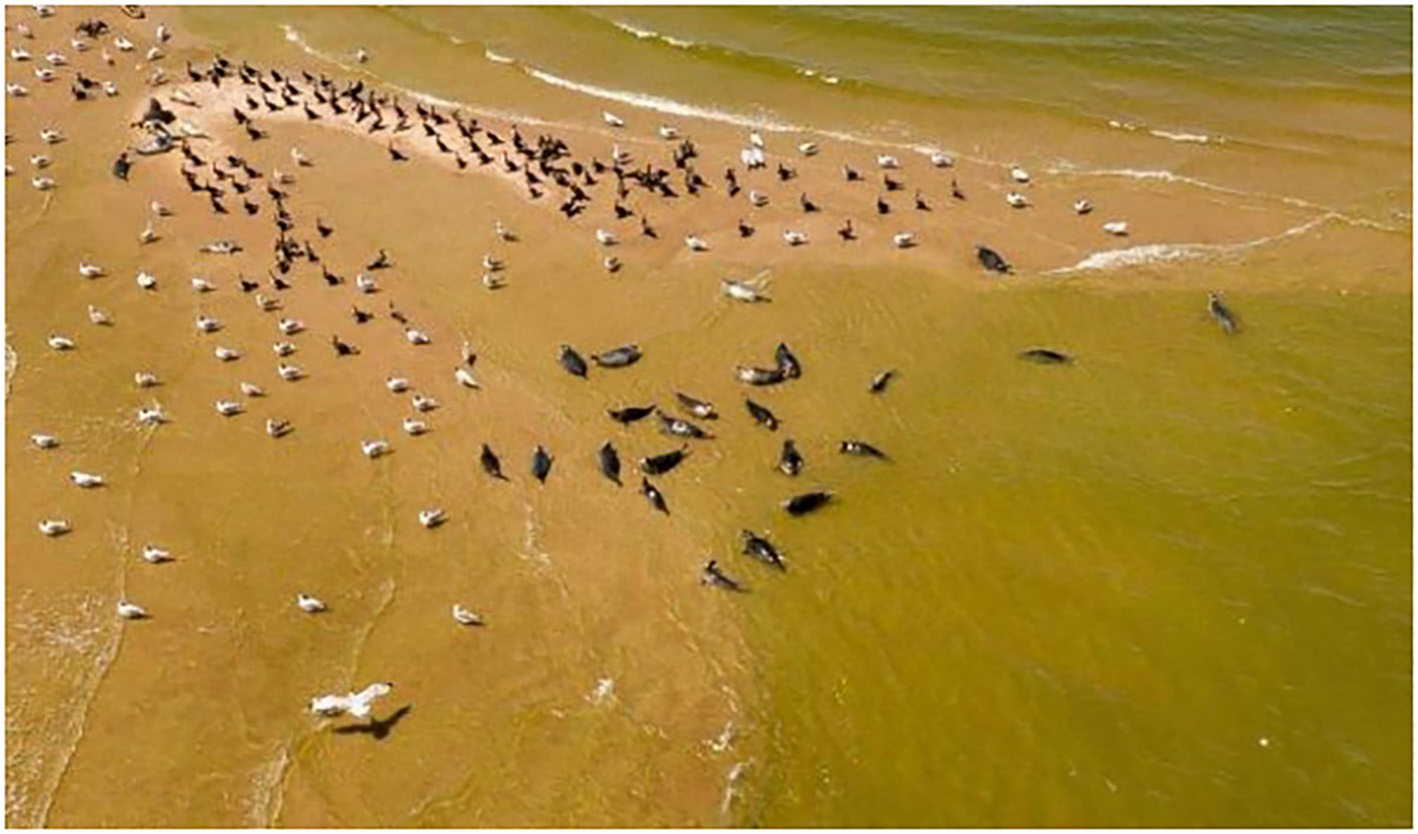
Northern tip of Maliy Zhemchuzhniy Island, northern Caspian Sea, Russia, showing Caspian seals in close proximity to aquatic birds (great cormorants, Caspian gulls and Caspian terns). Photo: ASNBR, 11 April 2020.

In May 2022, specialists from the Research Institute of Virology, Federal Centre of Fundamental and Translational Medicine, Siberian Branch, Russian Academy of Sciences (FRCFTM SB RAS), also investigated a considerable number of ill and dead Caspian terns (*Hydroprogne caspia*) and a lesser number of great black-headed gulls (*Larus ichthyaetus*), Caspian gulls (*Larus cachinnans*) and Dalmatian pelicans (*Pelecanus crispus*) on Maliy Zhemchuzhniy Island, a major intersection of migratory flyways north-east, north-west and south (Vilkov, 2016a; Veen et al., 2005) (**Figure 4**) and the major haul-out site of the Caspian seal in the Russian northern waters of the Caspian Sea (Krilov, 1976, 1982; Khuraskin and Pochtoeva, 1986) (**Figures 5**, **6**). This investigation was in response to notification by the Institute of Ecology and Sustainable Development (DSU) by Astrakhan State Nature Biosphere Reserve (ASNBS) authorities of this outbreak in the Maliy Zhemchuzhniy Island Specially Protected Natural Area of Federal Significance which they administer.

During the 20^th^ century, scientists characterized Maliy Zhemchuzhniy Island as a location where sick seals lingered after winter when healthy seals dispersed elsewhere in the Caspian Sea. Considerable research was undertaken in the later years of the USSR in documenting the gulls and terns frequenting the island and identifying viruses they carried (Andreev et al., 1980; Aristova et al., 1982, 1986). The island is the location of the largest breeding colony of great black-headed gulls documented as nesting in the Caspian region.

In the period April-May 2022, it was calculated that over 30,000 birds had died or were suffering on the island. According to ASNBR observations, some weakened birds stood and showed no fear on approach by humans, while others were losing coordination and tumbled. Their external neurological behavioral symptoms were similar to those of seals suffering from a highly pathogenic avian influenza virus strain (see further below). Diarrhea was the principal externally observable clinical symptom, although there was no abnormal discharge of oral mucosa. It was notable that dead birds had well-groomed feathers, indicating that each individual had rapidly succumbed to the virus. Among the terns and gulls nearly all chicks died during the nesting period: no live chicks were observed on the island later in the 2022 nesting period.

From samples taken from birds on the island in May 2022 and analyzed by the Research Institute of Virology (FRCFTM SB RAS), the H5N1 subtype was determined as the cause of the infections and is the outcome of sequential reassortment of multiple genetic variants of LPAI and HPAI viruses. The research indicates that in the fall of 2021, the HPAI virus moved along with migrating birds to their wintering sites, and in the winter of 2022 was detected in Israel. With the spring migration of 2022, it spread from the Middle East to nesting areas, causing the death of wild birds on Maliy Zhemchuzhniy Island (Sobolev et al., 2023).

**Figure 7:**
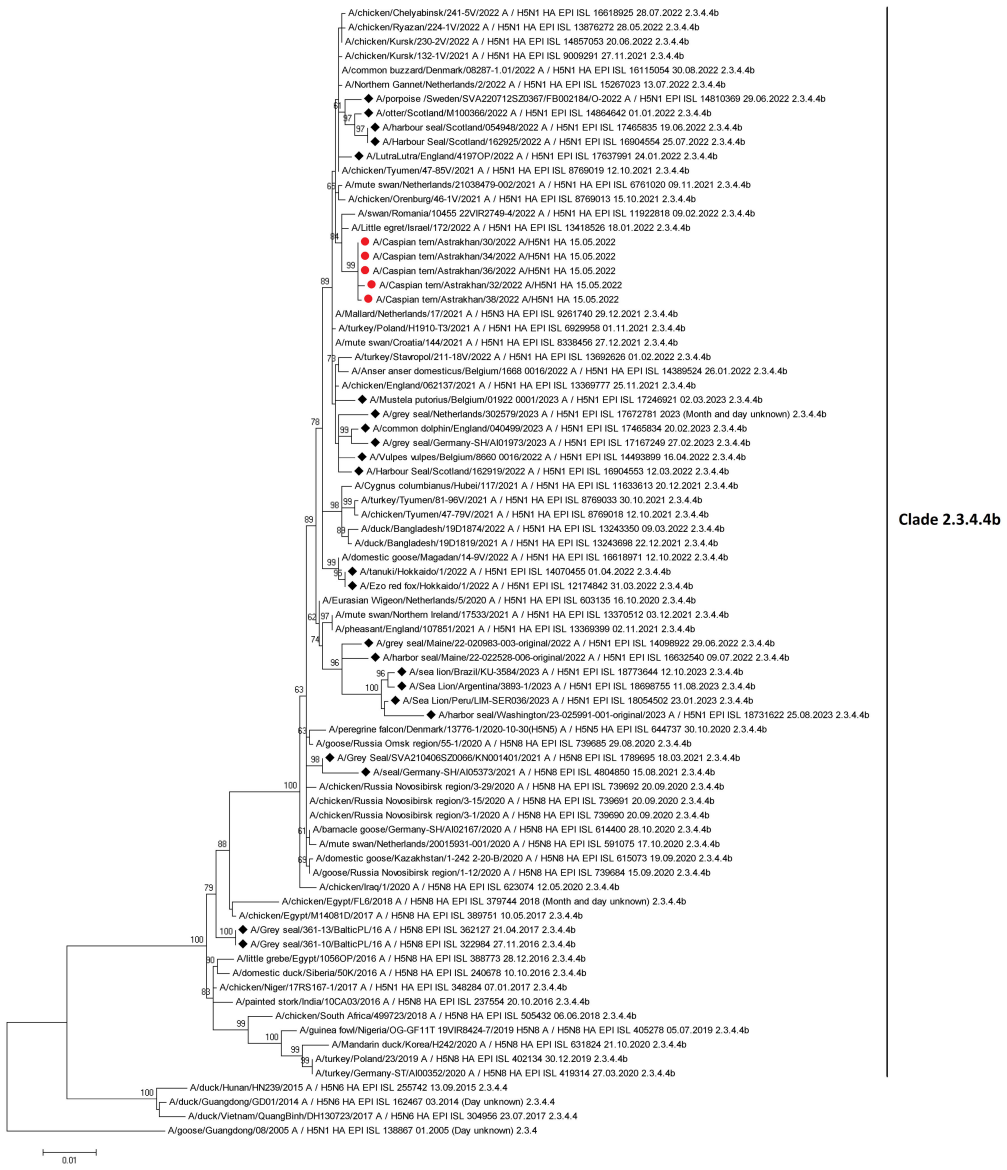
Maximum-likelihood phylogenetic tree of the hemagglutinin segment. Circles indicate HPAI H5N1 virus strains detected in birds on Maliy Zhemchuzhniy Island. Rhombuses indicate H5Nx viruses of clade 2.3.4.4b isolated from mammals (including marine mammals). The multiple alignment was performed using MUSCLE. Maximum-likelihood phylogenetic analysis was generated and visualized by MEGA5 using the general time-reversible nucleotide substitution model. Bootstrap support values were generated using 500 rapid bootstrap replicates.

There are currently 80 sequences of H5Nx influenza viruses isolated from seals and sea lions in the GISAID database, confirming the ability of the virus to infect these animals. Based on the results obtained by the authors of the article, as well as data posted in the GISAID database, we conducted a phylogenetic analysis for the HA gene of highly pathogenic influenza viruses found not only in birds, but also in mammals (**Figure 7**). According to the analysis of highly pathogenic H5N1 influenza viruses of clade 2.3.4.4b, isolated from mammals (including marine mammals), they are phylogenetically closely related to those circulating among birds, including those that caused mass deaths on Maliy Zhemchuzhniy Island. Thus, there is a possibility that when seals come into contact with aquatic avifauna infected with a highly pathogenic influenza virus, interspecific transmission of the pathogen is possible.

Here it should be noted that hundreds of deal Caspian seals were found on northern Dagestan shores in December 2023, clinically presenting similar symptoms to those sampled in the same region during the mass mortality of late December 2022.

## Other detections of HxNx strains in the Caspian region 2004-2023

Between 14-17 November 2005, an epizootic among mute swans occurred in the Russian sector of the lower Volga River delta. More than 250 dead swans were found in the Kamizyakskiy district, Astrakhan oblast and about 150 dead swans in the Laganskiy district of the Republic of Kalmykia. Samples from sick and recently died swans were analyzed by the D.I. Ivanov Institute of Virology, Russian Academy of Medical Sciences: ten HPAI H5N1 strains were isolated and deposited in the State Collection of Viruses of the Russian Federation (Lvov et al., 2006).

On 19 June 2022, two wild animals (species unidentified but probably waterbirds) were reported dead in north Caspian Sea waters off the Ural River delta near Atyrau, Kazakhstan. A report (Report ID FUR_157724) from the Kazakhstan authorities to the World Organization for Animal Health (WOAH) states that the cause was the H5 strain (N untyped) of avian influenza, as detected by the Republican State Enterprise National Reference Centre laboratory. Testing was done through polymerase chain reaction (PCR) (https://wahis.woah.org/#/in-review/4503?fromPage=event-dashboard-url). This identification was the latest in a series of Kazakh government laboratory AIV isolations from samples taken from water and shore birds along the Kazakhstan northern Caspian littoral in 2004 and later in 2006, as follows:

At the Institute of Microbiology and Virology, Almaty, Kazakhstan: A/great black-bed gull/Atyrau/743/2004 (H13N6)A/great black-headed gull/Atyrau/744/2004 (H13N6); A/great black-headed gull/Atyrau/767/2004(H13N6); A/great black-headed gull/Atyrau/773/2004(H13N6); A/common pochard/Aktau/1455/2006(H4N6); A/coot/Aktau/1454/2006(H4N6); and A/mute swan/Aktau/1460/2006(Y5N1) were identified; and A/swan/Mangystau/3/2006(H5N1) was identified at the Institute for Biological Safety Problems, Gvardeiskiy, Zhambyl Oblast (Bogoyavlenskiy et al., 2011). The latter was from a wild mute swan found dead on the coast of the Mangystau region in March 2006, the virus responsible being isolated and characterized by the Laboratory of Ecology and Viruses, Institute of Microbiology and Virology, Almaty and the Institute for Biological Safety Problems, Gvardeiskiy, Zhambyl Oblast. 70 wild birds were later found dead from H5N1 in this same area (Tabynov et al., 2008; Kydyrbaev et al., 2010). Phylogenetic analysis indicated that this subtype differs from EMA groups of HPAI H5N1 viruses and has NS2A NS2A genotype typical for Gs/Gd-like strains (Bogoyavlenskiy et al., 2011; Chervyakova et al., 2011). This led Chervyakova et al. to consider that this subtype may have been transmitted by birds from Europe (Sweden) along the Black Sea/Mediterranean avian flyways. These overlap with the Eurasia-Africa flyway in the Caspian region. See also Sayatov et al., 2007; Kydyrbayev et al., 2010; Bogoyavlenskiy et al., 2012; Tabynov et al., 2014; Burashev et al., 2020.

From the point of view of the evolution of avian influenza viruses and their transfer to other geographical regions, researchers of the Federal Centre of Animal Health, Vladimir, Russia (Zinyakov et al., 2022) have provided data for H5N5 viruses isolated from birds sampled in the northern Caspian region in 2020-2021 as follow: A/dalmatian pelican/Astrakhan/417-2/2021(H5N5) (recovered on 1 April 2021); A/dalmatian pelican/Astrakhan/417-1/2021(H5N5) (recovered on 1 April 2021); A/pelican/Dagestan/397-1/2021(H5N5) (recovered on 6 April 2021); A/gull/Dagestan/397-2/2021(H5N5) (recovered on 6 April 2021); A/waterfowl/Russia/1526-4/2021(H5N5) (recovered on 28 September 2021); A/shelduck/Kalmykia/1814-1/2021 (H5N5) (recovered on 8 November 2021), which the researchers have collectively referred to as Caspian Region Viruses (VCR). For these subtypes, a similar clustering of genomic segments was determined with isolates of the H5N5 subtype influenza A virus, which were identified in Europe in winter and spring 2021. Zinyakov et al. (2022) state that “phylogenetic analysis of nucleotide sequences of entire viral genomes belonging to the H5N5 subtype suggests that they all evolved through the reassortment of HPAI H5N8 with one unidentified N5 subtype virus. This was confirmed by the dense clustering of viruses carrying the HA and MP genes for H5N5 and HPAI of the H5N8 subtype, previously identified in the Russian Federation (an exception being the avian influenza virus A/eagle/Hungary/8569/2021).” H5N5 subtype isolates recovered in Europe in autumn 2020 were also included in the analysis. The results obtained indicated that the H5N5 subtype viruses detected in Europe and Russia in 2021 emerged in autumn 2020 (Zinyakov et al., 2022).

Laridae (gulls and terns in particular) constitute one of the principal reservoirs of AIVs. Their eco-geographic role in the global dynamics of the spread of AIVs, are significant but yet distinct from those of wild waterfowl (Kawaoka et al., 1988; Perkins and Swayne, 2002; Fouchier et al., 2005; Wille et al., 2011a; Ratanakorn et al., 2012; Tønnessen et al., 2013; Verhagen et al., 2014; Wille et al., 2014; Arnal et al., 2015; Benkaroun et al., 2016; Lang et al., 2016; Alkie et al., 2022, 2023; Lean et al., 2023). Although gulls are mainly vectors for the H13 and H16 subtypes, they have been shown to also be infected by other subtypes. They also often carry AIVs with mixtures of genes of different geographic phylogenetic lineages (e.g. EA and NA). A range of viruses detected in gulls and terns globally have been shown to possess very high phylogenetic affinities to viruses identified in other host taxa, thus demonstrating the potential for gulls to serve as HPAI virus carriers, which disseminate viruses across long distances and contribute to the genesis of pandemic variants. For instance, Huang et al. (2014) provide a chronology of the evolution of an entirely Eurasian gull virus (H16N3) which was isolated in North America. Genetic tracing indicates that the Caspian Sea was an important zone in the generation of this virus and that an analysis of AIVs isolated in certain tern species also indicates that the Caspian region plays in role in the generation and transmission of novel strains. Thus an analysis of the geographic and chronological progression of gull virus genes which contributed to the genesis of the Eurasian H16N3 gull virus isolated in Newfoundland, Canada, points to an origin in coastal Astrakhan in Russia in 1983, then in Scandinavia in 2006-2009, and then in Iceland and Newfoundland, Canada in 2010 (Huang et al., 2014; Benkaroun et al., 2016).

On 9 February 2023, the responsible Russian state agricultural authority, Rosselkhoznadzor, also reported that the North Caucasus Inter-Regional Veterinary Laboratory detected influenza A virus of subtype H5 in a sample taken from a dead swan (species unidentified) near Solnechnoe, Khasavyurtovskiy District, Dagestan, on 3 February 2023. This provided yet further evidence of the presence of avian H5 viruses in the region (unpublished internal report). A later report dated 14 February 2023 to the WOAH (Report ID FUR_159296) states that five mute swans (*Cygnus olor*) were found dead in that location on 12 January 2023, the Federal Centre for Animal Health (ARRIAH) reporting that the highly pathogenic avian influenza subtype had been detected through real-time reverse transcription polymerase chain reaction testing (https://wahis.woah.org/#/in-review/4846?fromPage=event-dashboard-url).

## Studies of Caspian seals for avian influenza A viruses: a chronology

The first studies of viruses in Caspian seals focused on AIV circulation, Yamnikova et al. reported negative results for embryonated chicken egg cultures of 152 samples collected from seals between 1976 and 1999 (Yamnikova et al., 2001; Kydyrmanov et al., 2023). Later, Ohishi et al. (2002) detected antibodies to H3N2, H2N2, H3N8, and influenza B viruses in Caspian seals. They reported that “antibodies to influenza A virus were detected in 54%, 57%, 40% and 26% of serum samples collected in 1993, 1997, 1998 and 2000 by enzyme-linked immunosorbent assay (ELISA). In a hemagglutination-inhibition (HI) test using H1-H15 reference influenza A viruses as antigens, more than half of the examined ELISA-positive sera reacted with an H3N2 prototype strain A/Aichi/2/68. These sera were then examined by HI testing with a series of naturally occurring antigenic variants of the human H3N2 virus and H3 viruses of swine, duck, and equine origin. The sera reacted strongly with the A/Bangkok/1/79 (H3N2) strain, which was prevalent in humans in 1979–1981. The results indicate that the human A/Bangkok/1/79-like virus was probably transmitted to Caspian seals in the early 1980s, and was circulated in the seal population. Antibodies to influenza B virus were detected by ELISA in 14% and 10% of serum samples collected from Caspian seals in 1997 and 2000, respectively.” The researchers concluded that the Caspian seal might be a reservoir of both influenza A and B viruses which had originated from humans (Ohishi et al., 2002).

These findings have been since supplemented by those of a collaborative research team from the Institute of Ecology and Sustainable Development (DSU) and the Research Institute of Virology (FRCFTM SB RAS), which isolated an avian-like H4N6 virus A(H4N6)T1 influenza A virus in a Caspian seal in Dagestan in 2012. The virus was considered to have probably originated from birds. The researchers emphasized that the role of pinnipeds “as intermediate hosts or carriers of potential zoonotic pathogens remains poorly understood (Fereidouni et al., 2014), highlighting a necessity for the long-term, systematic surveillance of influenza virus in marine mammals.” (Gulyaeva et al., 2021; Gulyaeva et al., 2018a, b).

After the widely-circulated reports of the mass mortality of seals in Dagestan in early December 2022, the Kaspika Caspian Seals Conservation Agency reported that specialists from the Biodiversity Conservation Service at the Ministry of Ecology and Natural Resources, Food Safety Agency and the Ministry of Agriculture of the Republic of Azerbaijan inspected the entire Azerbaijani coast of the Caspian Sea to identify possible seal mortalities and to determine the causes.

Seventeen dead seals were found by the team in the Khachmaz region (north Azerbaijan) and twenty-seven along the coasts of the Absheron Peninsula. Tissues and internal organs were sampled for toxicological, virological and bacteriological analyses. (http://kaspika.org/en/2022/12/14/monitoring-of-dead-seals-inazerbaijan-2/). The results have not yet been published.

## HPAI H5N1 deaths of northern fur seals and Stellar Sea lions and aquatic avifauna in eastern Russia and multiple wild avifauna fatalities due to the subtype in central and northern Russia 2023

In August 2023, the death of a northern fur seal (*Callorhinus ursinus*) in Mordvinov Bay on Sakhalin Island in the Sea of Okhotsk in eastern Russia was attributed by the Russian Federal Centre for Animal Health as due to HPAI H5N1 and reported to the WOAH (Report FUR_162532). Multiple (more than 300) deaths of northern fur seals and Stellar sea lions (*Eumetopias jubatus*) were also reported in early August 2023 by nature conservationists removing marine litter and pinniped entanglements on the uninhabited Tyuleniy Island off the east coast of Salkhalin Island. Samples from dead animals were later taken for analysis by specialists of the Federal Supervisory Natural Resources Service (livescience.com/animals/seals/mystery-mass-death-of-seals-or-remote-uninhabited-siberian-island-under-investigation). See also, Permyakov et al. (2023).

Throughout the summer months of 2023, multiple cases of Laridae (species unidentified) dying of HPAI H5N1 in the Komi Republic, Novgorod and Murmansk oblasts (central and northern Russia) and the Primorskiy Krai (eastern Russian seaboard) were also reported to the WOAH. For the latter region an epidemiological comment was appended that during the active monitoring of avian influenza in wild birds, one sample out of ten demonstrated the H5N1 avian influenza virus genome (https://wahis.woah.org/#/event-management Accessed 08.10.2023).

## Timeline of global (non-Caspian region) occurrences of seal mortalities attributed to avian influenza viruses and their clinical signs and pathologies

Historically, epizootics with considerable mortalities in seal populations have been documented mainly along the shores of the USA and Europe. Such deaths have been mostly attributed to viral infections (Siebert et al., 2010), increasingly caused in recent years by avian or avian-like influenza viruses.

Below is presented a global timeline of published AIV outbreaks or serological analyses with the geographical location of seals examined, the gross external clinical signs and symptoms of diseased seals, the subsequent laboratory analytical pathology undertaken and subtype designation as well as key associated conclusions.

**1979-1980:** The first influenza epizootics recorded in any pinniped species were of acute hemorrhagic and interstitial pneumonia in harbor seals (*Phoca vitulina*) along the Massachusetts (New England) coast of the USA. From 1979 -1980 some 600 harbor seals died – c. 20% of the estimated seal population in this region. Investigations revealed that these respiratory infections were caused by “an H7N7 influenza virus (designated subtype A/Seal/Mass/1/80), which was repeatedly isolated from the lungs, brain, and hilar lymph nodes of dead seals sampled.” (White, 2013)” See also Geraci et al., 1982 and Hinshaw et al., 1984.

Since the 1979-1980 outbreak, mortalities in harbor seal populations along the coasts of the United States and Europe have been largely attributable to viral infections (Siebert et al., 2010).

Subsequently, influenza virus infection has been evidenced through many influenza serotypes found through antibody and virus isolations from seal populations in many regions of the world. Serotypes of H5N1, H7N7, H4N5, H4N6, H3N8, and H3N3 have been isolated in harbor seals along the New England coast (White, 2013).

**1982-1984:** In 1981, no further increase in seal deaths or strandings was observed along New England shores. Between June 1982 and March 1983, more harbor seals were again found dead or dying of severe viral pneumonia along the New England Cape Cod coast (constituting a c. 2% to 4% mortality rate of the estimated regional seal population) (White, 2013). Although the pathology was similar, the strain of influenza A detected differed from that identified in the first 1979-1980 outbreak (Geraci et al., 1982; Hinshaw et al., 1984). From one sample from an emaciated adult seal stranded on Plum Island, Massachusetts in June 1982, Hinshaw et al. (1984) isolated an influenza A virus strain identified as H4N5, whose surface antigens had previously been detected only on avian viruses. These researchers observed that, “comparisons with other avian strains indicated that the hemagglutinin of this seal virus was closely related to isolates from ducks, e.g. A/Dk/Alb/686/82 (H4N6), and turkeys, e.g., A/Ty/Mn/28/78 (H4N8), and that the neuraminidase was indistinguishable from the prototype for N5, i.e. A/Shearwater/Aust/1/75 (H6N5). These findings indicated that this new virus strain found in seals, A/Seal/Mass/133/82, was most closely related antigenically to recent avian isolates and was clearly different from the previous H7N7 seal isolates recovered from 1979 to 1980” (Hinshaw et al., 1984). Later, Callan et al. (1995) described influenza A strains H3N3 and H4N6 isolated from harbor seals with interstitial pneumonia. Together these findings led to the conclusion that seals are indeed susceptible to influenza A viruses, which seem to have originated from avian reservoirs (H4N5, H7N7, H4N6 and H3N3) occurring in nature (Callan et al., 1995). Hinshaw et al. (1984) considered that, “this susceptibility may be an example of the adaptation of avian viruses to mammals, which would represent an intermediate step in the evolution of new mammalian strains.”

**1991-1992:** The monitoring of virus infection of seals was continued after the association of influenza A virus with pneumonia epizootics in seals off the New England coast in 1979-1980 and 1982-1983. In January 1991 and January to February 1992, influenza A viruses were isolated by Callan et al. (1995) from seals that had died of pneumonia on Cape Cod, Massachusetts. They reported that, “Antigenic characterization identified two H4N6 and three H3N3 viruses. This was the first isolation of H3 influenza viruses from seals, although this subtype is frequently detected in birds, pigs, horses and humans. Hemagglutination inhibition assays of the H3 isolates showed two distinct antigenic reactivity patterns: one more similar to an avian reference virus (A/Duck/Ukraine/I/63) and one more similar to a human virus (A/Aichi/2/68). The hemagglutinin (HA) genes from two of the H3 seal viruses showing different antigenic reactivity (A/Seal/MA/3911/92 and A/Seal/MA/3984/92) were 99.7% identical, with four nucleotide differences accounting for four amino acid differences. Phylogenetic analysis demonstrated that both of these sequences were closely related to the sequence from the avian H3 virus, A/Mallard/New York/6874/78.” Callan et al. (1995) further suggested that this indicates that influenza A viruses of apparent avian origin, including the H3 subtype viruses, had continued to infect seals. See also White, 2013.

Siebert et al (Siebert et al., 2010 - referring to Kennedy-Stoskopf, 2001) concluded “that the influenza A viruses that were isolated from North American seals between 1979 and 1992 were antigenically and genetically most closely related to avian influenza viruses, which in this particular biogeographical region suggests frequent spill-overs from pelagic birds.” They stated that, “no evidence for stable circulating seal-adapted lineages of influenza A viruses has been obtained so far.”.

Siebert et al (Siebert et al., 2010 – referring to Webster et al., 1981b; Geraci et al., 1982; Hinshaw et al., 1984). further reported that, “In experimental inoculation trials, all isolates from infected harbor seals in this region were able to replicate in ferrets, cats, pigs and phocid seals, including harbor, ringed and harp seals.” “Clinical signs of natural influenza infection were similar to those observed for Phocine Distemper Virus, which included dyspnea, lethargy, bloodstained nasal discharge and subcutaneous emphysema. Pulmonary lesions were predominantly characterized by necrotizing bronchitis and bronchiolitis and hemorrhagic alveolitis.” (Siebert et al., 2010 – referring to Geraci et al., 1982; Hinshaw et al., 1984).

**2011:** Again on the New England coast, in November 2011, 162 harbor seals were documented as having died of interstitial pneumonia, hemorrhagic alveolitis and necrotizing bronchitis pneumonia over a period of less than four months (four fold the expected approximate mortality rate in the region’s seal population). An H3N8 strain was isolated from several of the seals (Bodewes et al., 2015a, b). Antigenic and genetic analyses showed that all genes from each of the epizootic strains were of avian origin (Bodewes et al., 2015a, b; Lehnert et al., 2007; Munster et al., 2009; Pund et al., 2015; Bodewes et al., 2016; Hussein et al., 2016). Virus antigen and RNA was found in bronchiolar epithelium by immunohistochemistry (IHC) and in situ-hybridization (ISH) (Anthony et al., 2012; Yang et al., 2015).

Furthermore, a subsequent study by Mandler et al. (1990) demonstrated that the closest-matching avian strains to the 1980 H7N7 virus were from the same geographic region as the seal isolate. The H3N8 virus (A/harbor seal/New Hampshire/179629/2011) isolated from this outbreak demonstrated naturally acquired polymerase mammalian adaptation mutations, indicating that it is of interest for human public health (Hussein et al., 2016).

A number of pathological investigations on seals which later died during the 2014 northeastern European epizootic Seal/H10N7 outbreak (as outlined below), report similar necro-suppurative bronchopneumonial lesions with a bacterial co-infection (Zohari et al., 2014; Krog et al., 2015). This was a time during which the complete spectrum of virus-associated histological changes associated with viruses, viral antigen distribution and the target cell tropism of Seal/H10N7 in harbor seals were unknown. However, a 2016 publication by van den Brand et al. provides a more comprehensive description of histological, IHC, virological, bacteriological and parasitological findings in 16 naturally infected seals that were described previously by Bodewes and co-researchers (Bodewes et al., 2015a, b; Bodewes et al., 2016; van der Brand et al., 2016).

**2014-2015:** From spring 2014 to early 2015, some 3,000 harbor seals and grey seals reportedly died from avian influenza virus H10N7 in north-east Europe (Osterath, 2014). Initially, this subtype in seals was reported along the west Sweden and east Denmark coasts (Zohari et al., 2014; Krog et al., 2015), and then spread to seals off the coasts of west Denmark, Germany and the Netherlands (Common Wadden Sea Secretariat, 2014). It is estimated that over 10% of the population of these two seal species died in this outbreak (Bodewes et al., 2015a, b; Herfst et al., 2020).

At this time, Bodewes et al. (2015a) undertook histological, IHC, virological, bacteriological and parasitological examinations of 16 naturally infected seals that had been found to have a positive PCR for influenza A virus, as well as testing tracheal/throat swabs or lung tissue samples from a larger collection of dead or dying seals found along the German North Frisian coast and the island of Helgoland. They reported that, except for a single juvenile which was euthanized, all other seals had died of natural causes. All were reported to be in varying nutritional conditions, ranging from very poor to good. Gross findings in animals found dead comprised “poorly retracted lungs with severe congestion, alveolar and interstitial emphysema, alveolar edema, occasional diffuse consolidation, and multifocal firm nodular areas of grey-yellow discoloration with varying numbers of metazoan parasites. Other organs and tissues did not display significant changes.” (van der Brand et al., 2016).

**2016-2017:** In 2016 and 2017, during the HPAI H5N8 epizootic in Europe, an emerging, closely related subtype (clade 2.3.4.4b) was found in two grey seals on the Polish Baltic Sea coast (Shin et al., 2019). In November 2016, an emaciated, immature male grey seal was found dead on this coast in a condition of early decomposition. Pathologic findings by Shin et al. (2019) included a parasitic infestation of *Halarachne halichoeri* in the nasal cavity, lung, and gastrointestinal tract; agonal changes, including pulmonary edema and emphysema, were also documented.” A male pup, c. two months’ old, was then found in April 2017, also severely undernourished with signs of trauma and mild to severe parasitic infestation in its digestive tract. Analysis indicated the presence of several different bacteria. “Both seals were found to be infected by the same H5N8 virus (classified as H5N8/seal) with a multibasic cleavage site of PLREKRRKR/GLF in its HA protein, which fits the consensus sequence of a clade 2.3.4.b HPAI virus (WOAH, 2018). Phylogenetic analysis of the HA and NA segments further revealed that the isolate belonged to the clade 2.3.4.4b group of H5 HPAI viruses. Results of a homology BLAST search showed that this virus had a nucleotide homology of 99.7%–100% to AIVs viruses in circulation in wild aquatic avifauna in 2016 and 2017. This reassortant has been shown to be widespread and the cause of mass mortalities among waterfowl in many parts of the world, although no natural transmission from birds to marine mammals has yet been proven” (Shin et al., 2019).

In 2017, among AIVs which had already been identified in several seal species, evidenced by a pathogenicity spectrum from sub-clinical infection to mass mortality, an infection was identified by Venkatesh et al. (2020) in a 3-4-month-old grey seal pup, rescued from St Michael’s Mount, Cornwall, but which died shortly thereafter. The virus strain was revealed by nuceolitide sequencing to be of subtype H3N8. Through a GISAID database BLAST search and time-scaled phylogenetic analyses, the researchers inferred that. “this seal virus originated from un-sampled viruses, in local circulation in Northern Europe and likely to be from wild Anseriformes. On examining its protein alignments, several residue changes were revealed that did not occur in the avian viruses, including D701N in the PB2 segment, a rare mutation considered to be a characteristic of mammalian adaptation of avian viruses. Nevertheless, an avian influenza virus was not deemed to be the cause of death of the grey seal pup, as it had been reported to a rescue center after it was stranded, the AIV only being detected incidentally (Venkatesh et al., 2020).

**2020-2021:** Since autumn 2020 and following nearly 3 years of reduced AIV activity, Europe has been in the midst of another record-setting highly pathogenic avian H5Nx epizootic. However, unlike in previous events, increased signs are evident of avian flu spillover into mammalian species (European Food Safety Authority et al., 2021a, European Food Safety Authority et al., 2021b, European Food Safety Authority et al., 2021c). In 2020-2021 H5N1 infections were registered in wild birds and in more than 20 million farmed poultry in 28 European countries; effectively the most devastating HPAI epidemic that had ever occurred in Europe (Postel et al., 2022).

In Scotland from 2021 to early 2022, four dead seals (three harbor seals and one grey seal (found in Aberdeenshire, Highlands, Fife and Orkney) were tested positive for the HPAI H5N1 avian influenza virus through screenings undertaken by the Scottish Marine Animal Strandings Scheme (SMASS). At this time, the largest avian influenza outbreak in the British Isles had already spilled over into other mammals, about seventy having tested positive for the HPAI H5N1 subtype (https://www.gov.uk/government/publications/bird-flu-avian-influenza-findings-in-non-avian-wildlife/confirmed-findings-of-influenza-of-avian-origin-in-non-avian-wildlife). See also Baily (2014).

In mid-August 2021, Postel et al. (2022), screening for viral pathogens, investigated unusual influenza A virus infections in three dead adult seals found on the German North Sea coast. In their brain tissue, high virus loads of highly pathogenic avian influenza virus (HPAI) H5N8 were detected with the isolation of different virus variants indicating high exposure to HPAIV in circulation among wild avifauna. However, no regional evidence was found for H5 specific antibodies in healthy seals. It was thus suggested by these researchers that avian virus replication in seals may allow HPAIVs to acquire mutations needed to adapt to mammalian hosts, as shown by PB2 627K variants detected in these cases.

As outlined above, during a previous AIV (H10N7) outbreak in seals associated with pneumonia and slightly increased mortality, and in the context of an HPAI H5N8 infection in the lung tissues of two dead grey seals found on the Polish Baltic coast in 2016, the same researchers had found no evidence for any brain infection. “The HPAIV H5N8 strain currently in circulation is reported to cause unusual neurological infection with fatal outcomes in four harbor seals and one grey seal kept in a wildlife rehabilitation center. Similar to very recently reported findings in captive seals in the UK, these results show that the HPAIV H5N8 of clade 2.3.4.4b can induce fatal CNS infections in seals under natural conditions. The genetic findings underlined the role of seals as a possible “adaptive vessel” for avian influenza viruses and the importance of monitoring wild bird and mammal populations accordingly.” (Postel et al., 2022).

In September 2021, the Danish Veterinary Consortium’s Centre for Diagnostics (Statens Serum Institute) at the Technical University of Denmark detected the HPAI subtype H5N8 in a harbor seal found dead on the coast of Southwest Funen. This was the first time that highly pathogenic avian influenza virus had been found in seals in Denmark. The seal was emaciated with pronounced skin changes on large areas of the body of uncertain diagnosis. Influenza virus was detected in the lung but otherwise no other disease-causing organisms could be detected that could explain why the seal died. The Centre for Diagnostics examined 29 harbor seals and 15 grey seals in 2021 but this was the only one to test positive for an influenza virus.

The virus isolated in the seal from Southwest Funen was determined to be closely related to those causing outbreaks of avian influenza in wild birds and domestic poultry since the autumn of 2020, both in Denmark and in the rest of Europe. The Danish Veterinary Consortium performed a detailed analysis of the seal’s virus genome found some mutations that indicate possible adaptation to mammals, although it was not possible to analyze the full genome because of the decomposed state of the samples (https://outbreaknewstoday.com/denmark-reports-1st-highly-pathogenic-avian-influenza-case-in-harbor-seal-89870/).

In mid-2021, a novel reassortant influenza A(H10N7) virus was isolated from a dying adult male Pacific harbor seal washed ashore at Combers Beach near Tofino in British Columbia, Canada. The Animal Health Centre Laboratory in Abbotsford, British Columbia carried out its necropsy (Berhane et al., 2022), and reported it to be “in moderate body and fair postmortem condition. The notable findings from gross examination were that it had died from bronchointerstitial pneumonia with serosanguinous fluid within the thoracic cavity and focally extensive hemorrhage and edema from the skin to the visceral pleura along the right midlateral aspect of the thorax, with focal visceral to parietal pleural adhesion.” (Berhane et al., 2022).

“Histopathology revealed necrotizing bronchitis and bronchiolitis, peribronchiolar lymphoid hyperplasia, alveolar histiocytosis, and perivascular lymphoplasmacytic cuffing. Immunohistochemistry testing identified AIV antigen within the bronchiolar-associated lymphoid tissue of 2 bronchioles in more severely affected areas of the lungs and *in situ* hybridization confirmed AIV RNA. Aerobic cultures of the lungs, hilar lymph node, brain, intercostal skeletal muscle, and small intestine yielded variable mixed growth of *Streptococcus phocae* and *Serratia liquefaciens* with no fungal growth from the lung. PCR testing of pooled tissues proved positive for consensus influenza virus and mollicutes (Mycoplasma spp. were confirmed but could not be speciated) and negative for canine distemper virus.” (Berhane et al., 2022).

“The study of the seal showed that it had been infected with a novel reassortment H10N7 AIV containing a unique constellation of North American (NA) and Eurasian (EA) lineage gene segments and polymerase basic 2 segment D701N mutation, associated with adaptation to mammals. However, no conclusive evidence was found of where and when the reassortment occurred. However, the seal was recovered at a location within the Pacific Americas avian migratory flyway, where Gs/GD lineage H5N8 virus had been detected in 2014 and later reassorted with NA lineage viruses to create novel reassortant H5N2 and H5N1 AIVs that were responsible for large outbreaks in domestic poultry in Canada and the United States. The virus was designated as A/harbor seal/British Colombia/OTH-52-1/2021(H10N7).” (Berhane et al., 2022).

Berhane et al. also confirmed the observations of other researchers that seals share certain habitats with aquatic avifauna and that, therefore, the seal in this study may have been infected through viral spillover from infected birds.

Also in Canada in 2022, the Réseau québécois d’urgence pour les mammifères marins reported an unusually high number of dead or ill harbor seals in the St Lawrence River estuary, representing an increase of about 8 times the annual average of recent years. The results of field samples analyzed by the Quebec regional center (CQSAS) of the Canadian Wildlife Health Cooperative indicated that the increased deaths were attributable to infections caused by a highly pathogenic Eurasian H5N1 avian influenza virus. Further investigation by the molecular biology laboratory of the Ministère de l’Agriculture, des Pêcheries et de l’Alimentation du Québec indicated that this virus was present in most seal samples tested. Microscopic lesions characteristic of this viral infection were also observed in one of the seals autopsied. All positive cases came from the Bas-Saint-Laurent region (Blushke, 2022). No further information has been yet provided on clinical signs or pathological changes in the seals examined but Blushke hypothesizes that seals become infected following contact with infected seabirds (primarily via their feces and secretions) with which they share habitats.

The first detections of HPAI H5N1 clade 2.3.4.4b viruses in North America had already been found in wild and domestic birds in November 2021 in Newfoundland and Labrador in Canada and in late December 2021 in the United States (Ramey et al., 2022; Youk et al., 2023). It is believed that this virus was carried from Europe by seabirds (Shoham and Rogers, 2006; Pearce et al., 2009; Wille et al., 2011a; Van Borm et al., 2012; Hall et al., 2013, 2014; Huang et al., 2014; Bevins et al., 2016; Bevins et al., 2022; Boulinier, 2023; Caliendo et al., 2022; Kandeil et al., 2023; Lane et al., 2023). In Quebec, it has caused the death of several aquatic bird species, including snow geese, Canada geese, common eiders and northern gannets. Several raptor and scavenging species that feed on dead infected birds, such as turkey vultures, bald eagles, corvids and gulls, have also been affected. From January 2022 onwards, avian oropharyngeal or cloacal samples have been collected from wild birds in Massachusetts, USA: individual wild birds representing 78 avian species were evaluated as being of concern for H5 influenza and 119 infected birds from 21 species were identified (Puryear et al., 2023). HPAI H5N1 has also been detected in a number of North American land animals, including raccoons, striped skunks, red foxes, brown bears and Virginia opossums.

## Europe: mortalities of birds and land mammals caused by HPAI H5N1 2021-2023

Since 2005, when they first spread into Europe from Asia (Li et al., 2004; Lvov et al., 2004; Lvov et al., 2005a; Lvov et al., 2005b; Chen et al., 2005; Liu et al., 2005), HPAI A(H5N1) viruses have undergone extensive genetic diversification, including the formation of hundreds of genotypes following reassortment with other avian influenza A viruses (Centers for Disease Control and Prevention, 2023b, c). The clade 2.3.4.4b HPAI A(H5N1) virus has become widespread, causing record numbers of bird outbreaks in wild, backyard, village, and farmed birds. In 2022-2023, the H5NI avian influenza virus has continued to spread globally, prompting the EU’s European Centre for Disease Prevention and Control to characterize the 2021–2022 highly pathogenic avian influenza (HPAI) epidemic season as “the largest HPAI and most geographically extensive epidemic so far observed in Europe, including 3,573 HPAI virus detections in wild birds”. The epidemic has affected 37 European countries from the Svalbard islands to South Portugal and Ukraine, in some of which there has been a year-round persistence of influenza infection in indigenous wild birds, representing a change compared to the usual seasonal pattern in which infections cease during the summer months (European Food Safety Authority et al., 2022a, European Food Safety Authority et al., 2022b; European Food Safety Authority et al., 2023a; European Food Safety Authority et al., 2023b; European Food Safety Authority et al., 2023c).

A(H5) viruses were also detected in wild mammal species in Europe and North America and showed genetic markers of adaptation to replication in mammals. (https://www.ecdc.europa.eu/sites/default/files/documents/avian-influenza-overview-September-2022_0.pdf) & https://www.gov.uk/government/publications/avian-influenza-influenza-a-h5n1-risk-to-human-health/technical-risk-assessment-as-of-30-january-2023-for-avian-influenza-human-health-influenza-a-h5n1-2344b). In 2022, an outbreak at a coastal American mink farm in Spain was the first instance of HPAI H5N1 spreading within mammalian populations. Initially, two mink died from hemorrhagic pneumonia, with the H5N1 virus variant detected by RT-PCR. Although it was first suggested that the farmed mink had become infected after coming into contact with wild gulls which were able to access their food, subsequently it has been proposed that they may have been infected by contaminated raw poultry which they were fed (Parums, 2023; European Food Safety Authority et al., 2023a; European Food Safety Authority et al., 2023b; European Food Safety Authority et al., 2023c). It also appeared that the infection was spreading between the minks themselves (Aqüero et al., 2023). In June-August 2023, twenty-one fur farms in Finland also had animals infected by H5N1: arctic fox, silver/red fox, raccoon dog and American mink (European Food Safety Authority et al., 2023a; European Food Safety Authority et al., 2023b; European Food Safety Authority et al., 2023c).

Thus, there is now increasing concern that HPAI viruses detected in farmed and wild mammal species in Europe and North America in late 2022 are showing molecular adaptation markers for the ability to replicate in mammals (Parums, 2023; European Food Safety Authority et al., 2023a; European Food Safety Authority et al., 2023b; European Food Safety Authority et al., 2023c; Sidik, 2023).

In 2023, HPAI H5N1 virus infections in birds have continued to be widespread across Europe, the EFSA reporting that clade 2.3.4.4b viruses had seven genotypes, three of which have been identified for the first time during this time period, over the summer season. The EFSA also reported that H5N5 viruses continued to circulate among wild birds but only in northern parts of Europe (European Food Safety Authority et al., 2023a; European Food Safety Authority et al., 2023b; European Food Safety Authority et al., 2023c).

The March – April 2023 Avian Influenza Overview of the European Food Safety Authority (EFSA) European Centre for Disease Prevention and Control (ECDPC) and European Union Reference Laboratory (EURLAI) for Avian Influenza (EURLAI) observed that “the vast majority of reported HPAI virus detections in wild birds during this reporting period was among colony-breeding seabirds, particularly in gulls (family Laridae). Of the gulls identified to species level, nearly all were black-headed gulls, with a number of herring gulls. Regarding waterfowl, most of the HPAI virus detections were reported in mute swans, followed by greylag geese and pink-footed geese. More than 36% of the wild birds in which HPAI virus detections were reported, were not identified to species.” (https://efsa.onlinelibrary.wiley.com/doi/10.2903/j.efsa.2023.8039).

**Figure 8:**
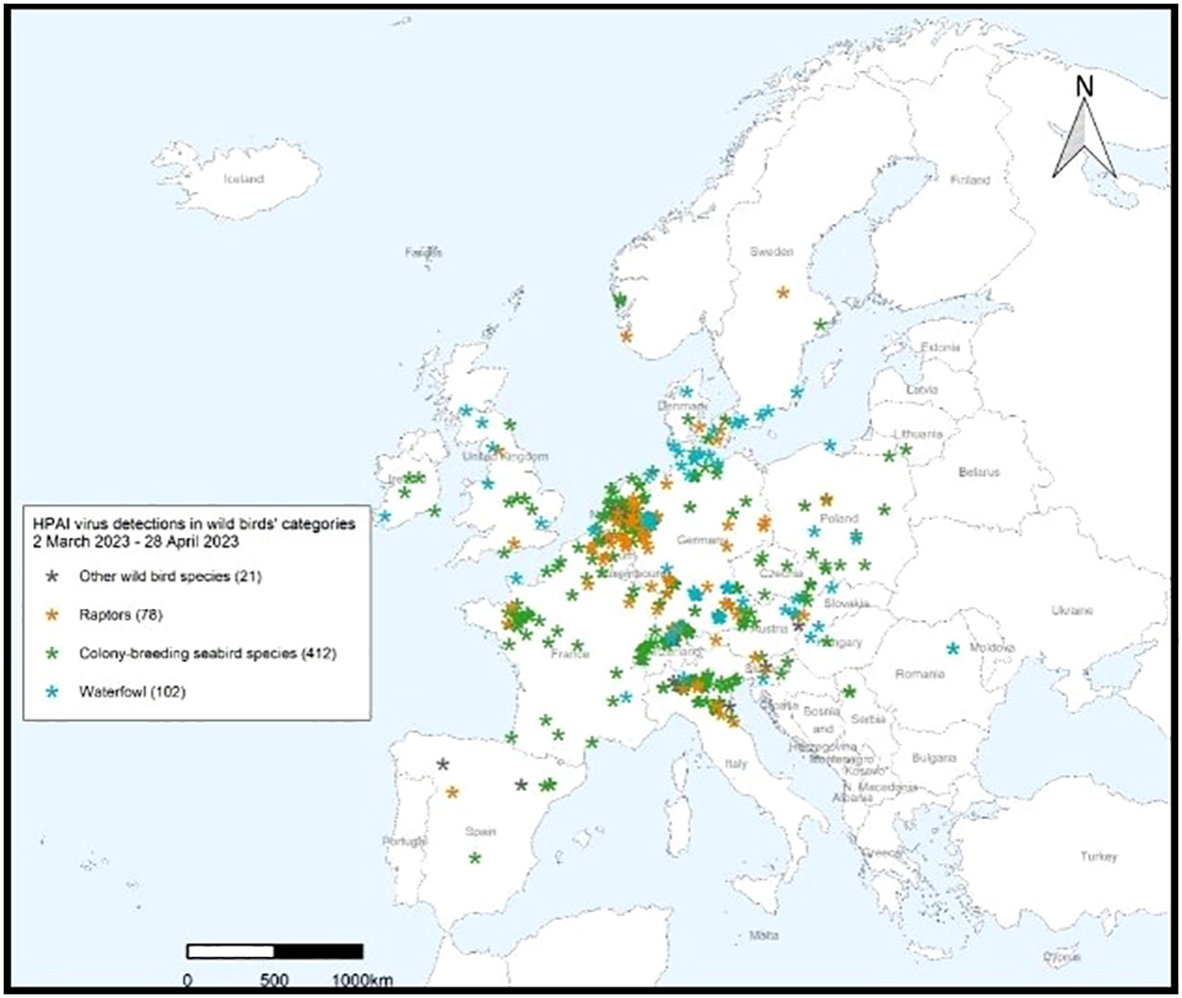
HPAI virus infections in wild birds’ categories 28 April 2023 – 23 June 2023. After European Food Safety Authority, European Centre for Disease Prevention and Control, European Union Reference Laboratory for Avian Influenza, Avian influenza overview April – June 2023.

**Figure 9:**
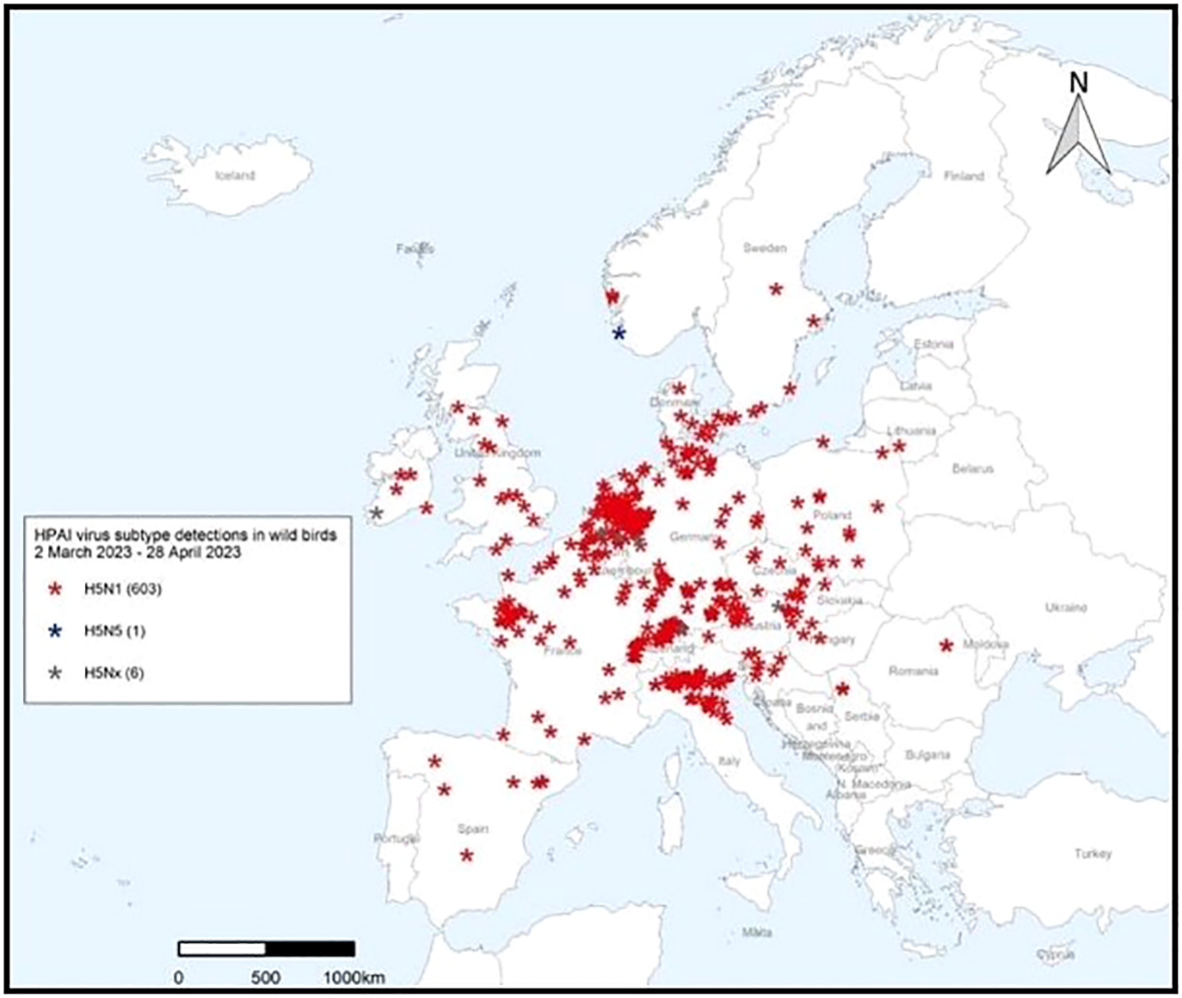
HPAI subtype detections in wild birds 29 April 2023 – 23 June 2023. After European Food Safety Authority, European Centre for Disease Prevention and Control, European Union Reference Laboratory for Avian Influenza, Avian influenza overview April – June 2023.

The April – June 2023 Avian Influenza Overview of the EFSA, the ECDPC and the EURLAI stated that “the A(H5N1) virus continues to circulate in breeding colonies of black-headed gulls. At the same time, as predicted, the host range had expanded to other gulls and terns, including Mediterranean gulls, black-legged kittiwakes, common terns and sandwich terns. Compared to the situation observed in 2022, seabird species were found dead not only along coastlines but also inland”. The overview advises that the increasing number of wild bird species involved in the A(H5) epidemic may alter the pattern of virus spread. Given the current geographical and temporal pattern of HPAI virus detections in wild birds in Europe and higher numbers compared to the same period in the previous year, The EFSA advised that it was expected that outbreaks in wild avifauna would continue to occur during the 2023 summer months (https://efsa.onlinelibrary.wiley.com/doi/epdf/10.2903/j.efsa.2023.8191) (**Figures 8**, **9**).

The overview also noted that the recent identification of A(H5N5) viruses of Eurasian origin in Canada and A(H5N1) viruses of African origin along the European Mediterranean coasts indicate the continuing inter-continental spread of the AIVs through wild bird migrations and that the actual number of wild bird deaths and the real impact of HPAI H5N1 is generally underestimated, as only very few are tested in diagnostic laboratories. The overview stated that, “A(H5N1) viruses currently circulating in Europe retain a preferential binding for avian-like receptors but that several mutations associated with increased zoonotic potential have been detected. Amongst mammals, wild and domestic carnivores continue to be the most affected mammalian species. The ongoing and Europe-wide HPAI A(H5N1) epidemic in seabirds represents a risk for marine mammals along the European coastlines, including harbor seals that breed in the Wadden Sea.” (European Food Safety Authority et al., 2023a; European Food Safety Authority et al., 2023b; European Food Safety Authority et al., 2023c).

In 2023, Mirolo et al. reported on H5N1 clade 2.3.4.4b infection closely related to sympatric avian influenza viruses, which they detected in the brain tissue of grey seals from the Dutch and German coasts in December 2022 and February 2023. Both these animals showed a “non-suppurative and necrotizing with viral antigen restricted to the neuroparenchyma. Viral RNA was also detected in the lung of the seal from Germany by real-time quantitative PCR. … No other organs tested positive. The mammalian adaptation PB2-E627K mutation was identified in approximately 40% of the virus population present in the brain tissue of the German seal. Retrospective screening for nucleoprotein-specific antibodies of sera collected from 251 seals sampled in this region from 2020 to 2023, showed no evidence of influenza A virus-specific antibodies. Screening by reverse transcription PCR of tissues of 101 seals that had died along the Dutch coast in the period 2020–2021, also did not show evidence of influenza virus infection. Collectively, these results indicate that individual seals are sporadically infected with HPAIV-H5N1 clade 2.3.4.4b, resulting in encephalitis in the absence of a systemic infection, and with no evidence thus far of onward spread between seals.”.

## North America: mortalities of land and marine mammals and birds attributed to HPAI H5N1 2021-2023

As stated above, the HPAI H5N1 clade 2.3.4.4b virus emerged in 2020 and was first recorded in November 2021 in eastern North America, subsequently being disseminated across the continent with significant adverse impacts on domestic poultry and widespread mortality in many wild bird species (Lenoch, 2023; Youk et al., 2023). In the United States alone, from May 2022 to June 2023, USDA APHIS (2023) reported HPAI A(H5N1) virus detections in 196 mammals of different species in 26 states or territories. The search for the reason/s underlying the spread of this subtype continues. One is that viral mutations may have facilitated its ability to replicate and thus infect a broader range of both bird and animal species (Parums, 2023; European Food Safety Authority et al., 2023a; European Food Safety Authority et al., 2023b; European Food Safety Authority et al., 2023c).

Puryear et al. (2023) in the most recent published study on a HPAI (H5N1) virus outbreak in seals reported on a further mass mortality in 2022 of New England harbor and grey seals that was concurrent with the second of two waves of wild bird AIV infections in the region. The first wave peaked in March and was largely represented by raptor deaths. The second wave began in June; gull and eider deaths being most frequently reported with mortalities in seabird breeding colonies throughout the coastal region (Puryear et al., 2023).

132 seals were found stranded along the North Atlantic coast from Maine to Virginia from January to July 2022. Highly pathogenic avian influenza virus was not isolated in seals that were sampled until the end of May 2022 concurrent with the first wave of avian infection but HPAI was found in samples collected after that during the second wave of June-July 2022, when in response to further seal strandings (164 harbor and 11 grey seals) in New England the National Oceanic and Atmospheric Administration declared an Unusual Mortality Event (UME) on June 1, 2022 (National Oceanic and Atmospheric Administration, 2010). 41 sampled (17/35 harbor and 2/6 grey seals) proved to be HPAI-positive and were within coastal regions where there had been known and suspected HPAI outbreaks among seabirds (Puryear et al., 2023).

The researchers observed viral sequences derived from both seal and avian hosts which reveal distinct viral genetic differences between the two waves. “Spillover into seals was closely related to the virus from the second wave and one of eight seal-derived sequences had the mammalian adaptation PB2 E627K.” (Puryear et al., 2023). Although, as noted above, harbor and grey seals had already been documented as being infected by the avian influenza A virus (in some cases involving seal-to-seal transmission), the 2022 outbreak was the first-known population-scale mammalian mortality event associated with the emerging avian influenza H5N1 clade 2.3.4.4b.

The researchers reported, “respiratory symptoms … with a subset of neurologic cases, although most stranded seals sampled were deceased. The respiratory tract was the most consistent source of PCR–positive samples from affected seals. Transmission from wild birds to seals was evident for >2 distinct HPAI H5N1 lineages in this investigation and likely occurred through environmental transmission of shed virus. Viruses were not likely acquired by seals through predation or scavenging of infected animals, because birds are not a typical food source for harbor or grey seals.” It was further concluded that,” data do not support seal-to-seal transmission as a primary route of infection. If individual bird–seal spillover events represent the primary transmission route, the associated seal UME suggests that transmission occurred frequently and had a low seal species barrier. Novel amino acid changes were also throughout the virus genome in seals, including amino acid substitutions associated with mammal adaptation.” (Puryear et al., 2023).

On 1 September 2023, in the USA the Washington State Department of Health and the Washington Department of Fish and Wildlife advised that there was an outbreak of HPAI among wild birds and seals with the deaths of more than 1,700 Caspian terns and gulls and at least 3 harbor seals on Marrowstone Island in Puget Sound. Infected Caspian terns had also been recently found near Port of Everett, Port of Tacoma and long the lower Columbia River (wdfw.wa.gov/species-habitats/diseases/bird-flu).

**Figure 10:**
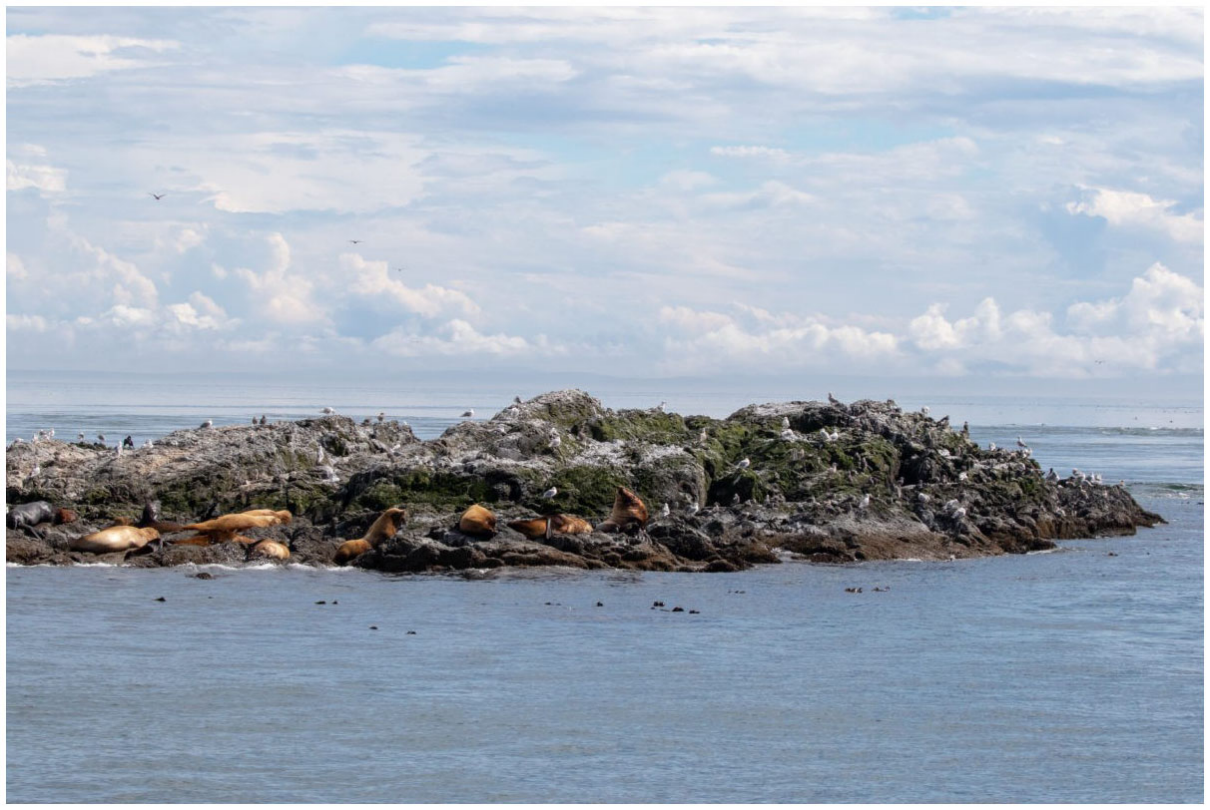
Steller sea lions (a threatened species) and aquatic birds (mainly glaucous-winged gulls – a vector for AIV) in typical close habitat association on Whale Rocks, WA, USA. Used by permission of the San Juan Preservation Trust (*sjpt.org*). Photo by Stephanie Colony.

The US Department of Agriculture National Veterinary Services Laboratory has since confirmed the H5N1 subtype in the three adult harbor seals found stranded. This has serious implications for pinniped species and birds inhabiting the Pacific coasts of the USA and Canada whose ranges stretch into the Aleutian Islands, a lengthy littoral zone utilized as the Pacific Americas migratory flyway (**Figure 10**).

## Central and South America and Antarctica: mortalities of marine mammals and wild birds 2022-2023 due to H5N1

The geographical extent of the ongoing H5N1 epidemic has been highlighted by infections rapidly carried by North American birds migrating to Central and South America, the avian influenza virus entering the continent through Colombia and thence Ecuador, Peru, Chile, Venezuela, Argentina, Uruguay, Brazil, Bolivia, Costa Rica, Honduras, El Salvador, Mexico and Cuba. It is currently occurring in both wild birds and poultry (Pan American Health Organization, 2023). HPAI outbreaks in so many countries in Latin America and the Caribbean had never recorded before. They are mainly located in areas of the Pacific Americas migratory flyway, the Peruvian pelican being the most frequently detected infected wild bird species with thousands of deaths being reported. The most affected mammal is the South American fur seal.

Ecuador declared an animal health emergency at the end of November 2022 and issued an epidemiological alert in December. Ecuador’s Ministry of Health confirmed the first case of avian influenza human transmission (with minor symptoms) in Bolivar province. This was the first human infection recorded in South America, a month after the country declared the emergency.

In mid-November 2022, Peru confirmed the furthest south occurrence of HPAI H5 into South America to that date. Several Peruvian pelicans tested positive on Los Cangrejos beach in Paita, well south of the first two South American (Colombian) outbreaks reported in late October 2022. By the end of November, the government of Peru had reported thousands of dead birds (mostly pelicans), and that they were investigating the suspicious deaths of several South American sea lions.

Samples were analyzed by the veterinary pathology laboratory of the Universidad Nacional Mayor de San Marcos. The initial results obtained were positive for highly pathogenic avian influenza H5N1 for South American sea lions, dolphins, Humboldt penguins, Peruvian pelicans, boobies and oystercatchers and since then have included cormorants, Dominican gulls, Peruvian gulls, grey gulls, Franklin gulls and red-headed vultures. See also Heckel and Schramm (2021).

**Figure 11:**
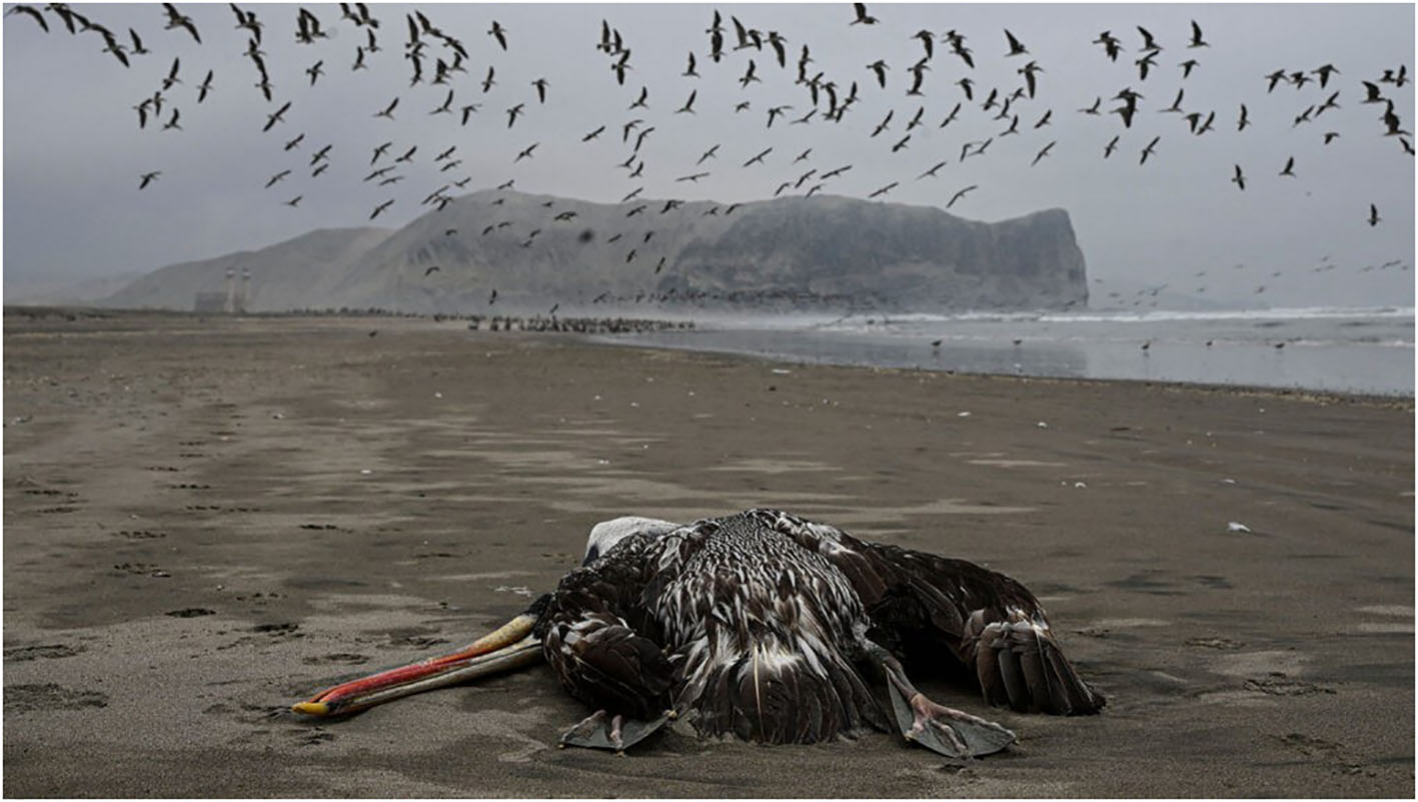
Peruvian pelican stricken with HPAI H5N1 on the Peruvian coast. South American sea lions in the background. February 2023. After: https://phys.org/news/2023-02-peru-hundreds-sea-lion-deaths.html.

By the first week of February 2023, 630 South American sea lions and 4 South American fur seals had died with the H5N1 strain detected in the tissues of 6 sea lions sampled and some 555,000 wild birds were reported as dying of H5N1 avian influenza in state coastal and marine protected areas, according to laboratory analyses undertaken by SENASA, the national agricultural health agency, prompting the declaration of a 180-day “biological vigilance protocol” (**Figure 11**). (https://phys.org/news/2023-02-peru-hundreds-sea-lion-deaths.html).

By March 2023, at least 3,487 South American sea lions had been found dead in Peru in association with the HPAI H5N1 outbreak, amounting to 3.3% of the estimated total population in the country and five times as many as previously reported.

Lequia et al. (2023), in a study of HPAI A/H5N1 affecting wild aquatic avifauna and marine mammals in South America, found, “that all Peruvian viruses belong to the HPAI A/H5N1 lineage 2.3.4.4b, but they are 4:4 reassortants where 4 segments (PA, HA, NA and MP) positioned within the Eurasian lineage that initially entered North America from Eurasia, while the other 4 genomic segments (PB2, PB1, NP and NS) position within the American lineage (clade C) that was already circulating in North America. These viruses are rapidly accruing mutations as they spread south. Peruvian viruses do not contain PB2 E627K or D701N mutations linked to mammalian host adaptation and enhanced transmission, but at least 8 novel polymorphic sites warrant further examination.” The HPAI outbreak in Peru has also been the subject of studies by Youk et al. (2023) and Gamarra-Toledo et al. (2023a, b). See also Blanc et al. (2009), Ministerio de Salud del Peru (2022), and Ministerio de Salud del Peru (2023).

Still further south, in November 2022, Chile started recording its first cases of avian influenza after 20 years during which no HPAI infections were recorded. The H5N1 variant was found in two Peruvian pelicans, the Chilean Agriculture Ministry reported, one in the coastal region of Iquique and another in coastal Antofagasta. In February 2023, it was diagnosed in a juvenile kelp gull in the Los Lagos region and has since also been found in Humboldt penguins (https://www.reuters.com/business/healthcare-pharmaceuticals/chile-confirms-new-cases-bird-flu-pelicans-2022-12-09/), as well as South American sea lions (H5N1 virus was detected in 6 of 6 sea lions), South American fur seals and marine otters.

By August 2023, the Chilean Agricultural and Livestock Service, had detected the presence of cases of HPAI H5N1 in 16 regions of the country in 871 wild birds (50 species), while the Servicio Nacional de Pesca y Acuicultura (SERNAPESCA) had detected avian influenza A(H5) in 47 aquatic mammals (South American sea lion, South American fur seal, Chilean dolphin, sea lion, spiny porpoise, Humboldt penguins, and Southern river otter) in 13 regions of the country, the majority of cases concentrated in the Northern zone, although H5 has also been detected among South American fur seals in Puerto Williams in the far south of Chile (see below).

The detection and phylogenetic analysis of H5N1 clade 2.3.4.4b in Chile in 2022 is the subject of a study by Jimenez-Bluhm et al. (2023). For the situation amongst marine mammals in South America see Gamarra-Toledo et al. (2023b).

**Figure 12:**
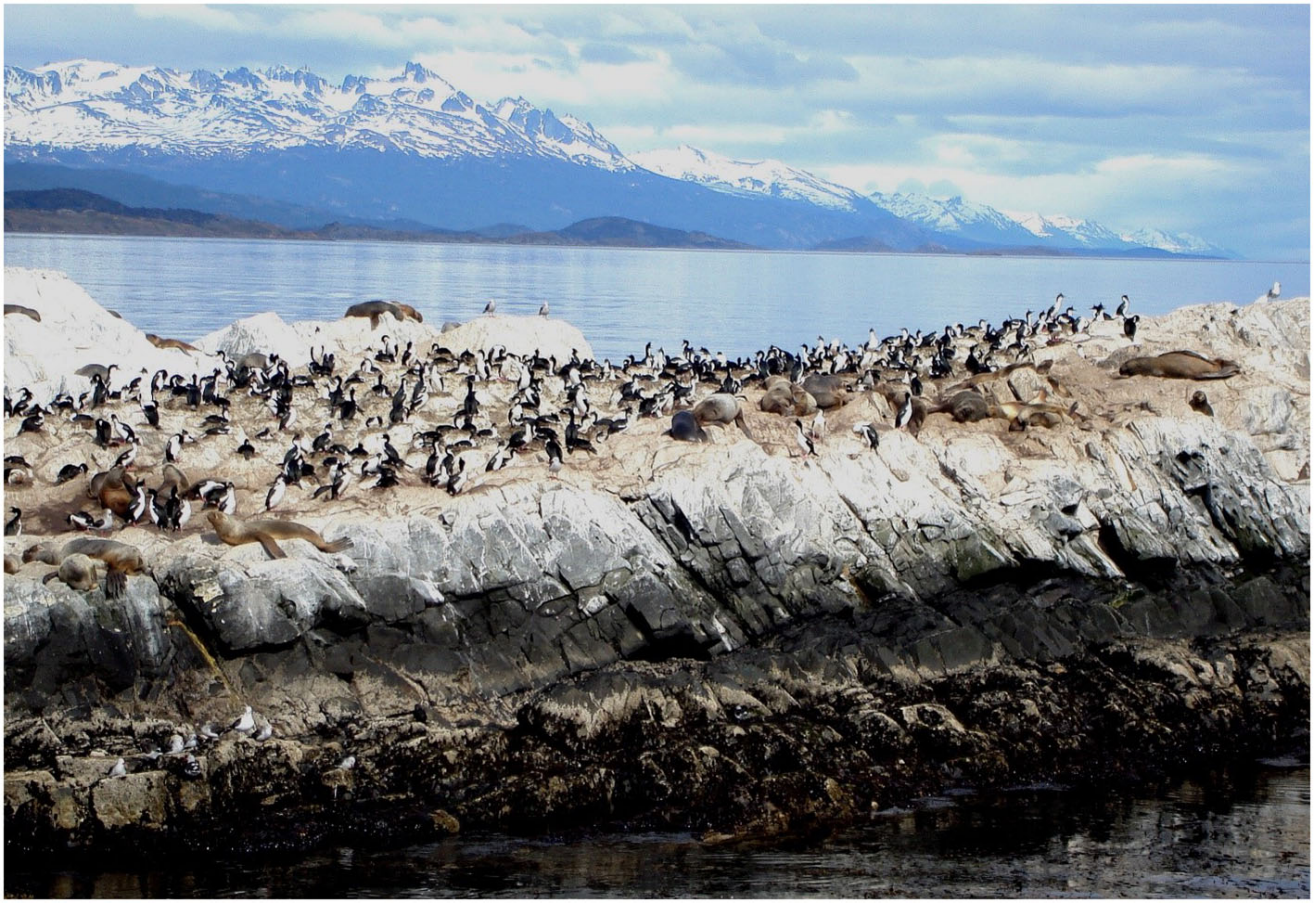
South American sea lions and aquatic birds (rock cormorants and Dominican or dolphin gulls) sharing the same habitat and in close association. Beagle Channel, Tierra del Fuego, Argentina. October 2007. Photo: gailhampshire from Cradley, Malvern, U.K, CC BY 2.0 https://creativecommons.org/licenses/by/2.0 via Wikimedia Commons.

The first occurrence of the H5N1 virus detected in Argentina was in an Andean goose in February 2023. In August 2023, 27 South American sea lions were found dead within the port of Necochea, Buenos Aires. Testing for avian influenza virus was undertaken by the Servicio Nacional de Sanidad y Calidad Agroalimentaria (SENASA). Due to the investigations and collateral damage that could be generated in the case of a zoonosis, the Argentine Naval Prefecture closed transit to the area. Further south in Argentina, 26 South American sea lions also died from avian influenza in Rio Grande, Tierra del Fuego. (**Figure 12**) 2 sea lions exhibiting neurological symptoms compatible with HPAIV died in Puerto Loyola, Santa Cruz as did 2 other sea lions also in the Reserva Faunística Punta Bermeja (La Loberia), Rio Negro. In all, by the third week of August 2023, SENASA reported to WOAH that 3 South American fur seals and 715 South American sea lions had died in 16 locations along the coast of Argentina. Analyses indicated that the cause of death was HPAI H5 (N untyped). https://www.mdzol.com/sociedad/2023/8/22/investigan-la-muerte-de-lobos-marinos-por-un-posible-brote-de-gripe-aviar-362729.html & https://wahis.woah.org/#/in-review/5189.

In September 2023, the Galapagos National Park Directorate (Ecuador) advised that three birds on the Galapagos Islands had been found to be infected with HPAI H5NI. This puts the endangered (IUCN) Galapagos fur seal at potential risk. (https://www.jpost.com/health-and-wellness/article-759776 Accessed 30.09.2023)

In Brazil, the first case of HPAI H5N1 infecting marine mammals (South American sea lions and South American fur seals) was reported on 1 October 2023 on Cassino beach, Rio Grande do Sul. This was followed by a second infection of the same two pinniped species in the same province. Samples were collected in partnership with the regional Marine Animal Recovery Centre and were analyzed by the Laboratório Federal de Defesa Agropecuária in Campinas (https://g1.globo.com/economia/agronegocios/noticia/2023/10/07/brasil-registra-2-foco-de-gripe-aviaria-em-mamiferos-marinhos.ghtml Accessed 10.10.2023). By 11 October 2023 the deaths of a further 31 sea lions and 23 fur seals due to H5N1had been reported (WOAH Report FUR_163252). The death of Magellanic penguins was also reported (European Food Safety Authority et al., 2023a; European Food Safety Authority et al., 2023b; European Food Safety Authority et al., 2023c).

Also in the first half of October 2023, the deaths of some 400-800 South American sea lions were reported in Uruguay and attributed to HPAI H5 (untyped) by the Dirección Nacional de Recursos Acuáticos. Also 43% of sea lions tested in various coastal colonies proved positive for H5, and it was proposed that transmission of the virus occurred when sea lions ate infected dead or sick birds (https://en.mercopress.com/2023/10/12/avian-flu-uruguay-admits-burying-400-sea-lions-but-a-similar-number-tested-positive Accessed 18.10. 2023). The death of Magellanic penguins was also reported in mid-2023 European Food Safety Authority et al., 2023a; European Food Safety Authority et al., 2023b; European Food Safety Authority et al., 2023c). Marandino et al. in 2023 published a detailed study on the spread of clade 2.3.4.4b of H5N1 in Uruguay (Marandino et al., 2023).

Following the detection in August 2023 of HPAI A(H5) in a South American sea lion in Puerto Williams (Chile) in the far south of South America (European Food Safety Authority et al., 2023a; European Food Safety Authority et al., 2023b; European Food Safety Authority et al., 2023c), yet further south within the broader Antarctic region, the H5N1 clade 2.3.4.4b AIV subtype (confirmed by the British Animal and Plant Health Agency) has been detected from September 2023 onwards by researchers of the British Antarctic Survey in brown skuas and in a southern giant petrel on Bird Island, part of the British overseas territory of South Georgia and South Sandwich Islands (https://www.sciencealert.com/lethal-form-of-bird-flu-found-in-the-antarctic-for-the-first-time Accessed 28.10.2023). Bird Island is c. 600 km south-east of the Falkland Islands (where H5 was detected in a southern fulmar in late October 2023 and then in a grey-backed storm petrel and in Falkland steamer ducks) and one of the nearest islands to the Antarctic landmass. Infected kelp gulls were also identified in three other locations in South Georgia in late 2023.

In the islands of the British Antarctic Territory brown skuas and kelp gulls scavenge and prey on young in dense penguin nesting colonies and scavenge among pinnipeds in their haul-out sites. Solitary leopard seals can be seen patrolling the shore to catch penguins as they enter or leave the sea during the breeding season. (Petherbridge, field observation Falkland Islands, British Antarctic Territory). Thus there is the potential for skuas to transmit the H5N1 virus to penguins which, in turn, could be infective prey for predatory leopard seals. Leopard seals, scavenging kelp gulls and brown skuas, pelagic birds (e.g. southern giant petrels, snowy sheathbills) and migratory birds could be carriers of the virus further south to Antarctic mainland bird species (penguins and albatross) (Baumeister et al., 2004). The six Antarctic pinniped species are also vulnerable: Antarctic fur seals, Ross seals, southern elephant seals, crabeater seals and Weddell seals. Here it also should be noted that various LPAIs have already been detected in a number of Antarctic region bird species since as early as 1981 (Bennison et al., 2023).

Following the detection of H5N1 in avifauna on Bird Island, a mass mortality of southern elephant seals was recorded elsewhere on South Georgia, a number exhibiting characteristic symptoms of HPAI in pinnipeds, such as difficulty breathing, coughing and accumulations of mucus in the nasal area. Many South American fur seals were similarly affected. Lethargy, neck spasms, twitching and an inability to fly were characteristic of H5N1 clinical presentations observed in brown skuas and kelp gulls (Bennison et al., 2023). Here it should be noted that similar symptoms have been recorded in avifauna infected with H5N1 in other parts of the world, including among sea birds in the northern Caspian Sea region (Sobolev et al., 2023), just as the infected elephant seals in South Georgia displayed similar external symptoms to those observed in 2022 in the northern Caspian.

A comprehensive breakdown of the current status of H5N1 in South America (as of 23 December 2023) has been provided by the OFFLU (WOAH-FAO Network of Expertise on Animal Influenza) ad-hoc Working Group on HPAI in Wildlife of South America and Antarctica (2023). For the Antarctic region, see also Dewar et al., 2023 of the Scientific Committee on Antarctic Research for an evaluation of the biological risk of highly pathogenic avian influenza in the Southern Ocean, as well as Wille et al. (2024).

On 19 January 2024, the Scientific Committee on Antarctic Research (SCAR) reported on its official website the presence of avian influenza (H5N1) in Gentoo penguins living on the Falkland Islands following the death of 35 penguins. As of 30 January, over 200 penguin chicks were reported to have died or were dying due to the virus.

On 24 February 2024, it was confirmed by the Spanish Higher Council for Scientific Research that researchers from the Severo Ochoa Biology Centre, based at Spain’s Antarctic research base, Gabriel de Castilla on Deception Island, had isolated the H5N1 virus from samples from two dead brown skuas that Argentinian scientists had collected near their research base, Primavera, on the northern tip of Antarctica’s mainland.

## Western and Southern Africa: recent mortalities of marine mammals and wild birds 2021-2023

In Africa H5Nx viruses have been detected amongst wild birds and poultry in Algeria, Benin, Burkina Faso, Cameroon, Cote d’Ivoire, Gambia, Ghana, Lesotho, Mali, Mauritania, Niger, Nigeria, Senegal, South Africa and Togo (Letsholo et al., 2022). A diverse range of H viral genotypes responsible were isolated, including H5N8, H5N6 and H5N1 (Bruno-Ouoba et al., 2022; see also Zecchin et al., 2017; Fusaro et al., 2019; Ndumu et al., 2018; Hamoudi et al., 2020; Makalo et al., 2022; Molini et al., 2022; Peyrot et al., 2022; Beyit et al., 2023 for a detailed general account of the dynamics of Hx viral impacts in Africa to that date), before H5N1 viruses emerged in many African territories between 2019 and 2021, with the most rapid rate and extent of spread being in 2021. The primary routes of transmission of H5N1 into the African continent have been inter-continentally along the Black Sea-Mediterranean and West Asian-East African migratory flyways and intra-continentally by aquatic avifauna migrating seasonally between western and southern Africa.

Since 2017 there have been multiple reports from South Africa’s Onderstepoort Veterinary Institute to the WOAH of HPAI H5 infections in that country, mainly among aquatic birds (including far-ranging pelagic species) (https://wahis.woah.org/#/event-management Accessed 08.10.2023). See also Cummings et al., 2008.

In April 2021, the HPAI H5N1 outbreak spread to South Africa as reported to the WOAH. The West Cape provincial government noted particular concerns for endangered (IUCN) Cape cormorants. The first outbreaks (transmitted by wild avifauna) were also reported at about this time in Lesotho and Botswana. The most comprehensive recent report of H5N1 among avifauna in southern Africa is by Abolnik et al. (2023).

In 2019, the Central Veterinary Laboratory of Namibia reported to the WOAH an outbreak of HPAI H5N8 among critically endangered African penguins. In both South Africa and Namibia there were also mass mortalities of Cape fur seals. However, this was not attributed to avian influenza infections but to malnutrition caused by drastic commercial overfishing of feeding grounds. As a prophylactic measure against transmission of HPAI to its wild birds and its vast population of Cape fur seals, the Namibian government (energized by the dedicated work of Ocean Conservation Namibia) in 2022 banned imports of poultry and poultry products from Denmark and the Netherlands where HPAI H5N1 outbreaks had been detected.

In January 2023, the WOAH was advised of the first occurrence in Namibia of the H5N1 subtype. This was among endangered Cape cormorants. In March 2023, outbreaks of HPAI were detected in great white pelicans in Mauritania. In April 2023, there was an unusual mortality recorded in wild birds in Tanji Bird Reserve in Gambia, as well as in Senegal, which was attributed to HPAI H5N1. WOAH also reported H5N1 in 752 wild birds (tern, pelican, cormorant and vulture species) in the Kapatchez region, Guinea-Bissau, West Africa. In mid-2023 the A (H5) virus was detected in Nigeria among several wild bird species, including greater crested tern, pied crow and grey crowned crane (European Food Safety Authority et al., 2023a; European Food Safety Authority et al., 2023b; European Food Safety Authority et al., 2023c).

Since the spread of the H5N1 subtype to southern Africa, no mortalities attributable to it have yet occurred among the sole pinniped species endemic to the south-west African coast, the Cape fur seal, in spite of its large populations there and higher mortalities among African penguins, Cape cormorants and other seabirds (including terns) in the region in 2021-2022 than in 2018-2019.

## Discussion

From the present overview it is clear that pinnipeds are susceptible to certain avian A influenza virus strains of high pathogenicity. At present the H5N1 strain (and particularly its Clade 2.3.4.4b) is having a severe impact on pinniped species across a wide geographical range globally. It is also clear that avifauna, in particularly water and seabirds of the Anseriformes and Charadriiformes orders found in sympatric habitat association with pinnipeds, are the principal vectors for the transmission to them of HPAI AIVs.

Although knowledge is growing into the phylogenetics of the viruses concerned and the dynamics of actual viral infection within a host (interactions between host factors and a virus being crucial for viral infectivity and host responses), it is also clear that the actual physical-chemical factors which facilitate viral transmission in shared environments between bird to bird, bird to mammals and among pinnipeds themselves require further exploration. Factors of significance may include: salinity, pH, temperature and chemical and biological constituents of aqueous environments/habitats; length of exposure to environment in natural conditions, contact with feces and other bodily secretions, etc. Additionally, among pinnipeds infection may occur through close intimate contact (including biting, aerosol transmission) at haul-out sites.

The evidence of research into the geographical spread of the HPAI H5N1 clade 2.3.4.4b over the past two decades clearly indicates the particular role played by wild bird vectors migrating through the northern Caspian Sea littoral. This region constitutes a critical global hub in the spread of highly pathogenic avian influenza viruses along flight paths to central and northern Europe and thence across the Atlantic Ocean to North America (and back again) and ultimately Central and South America, affecting birds, land mammals and pinnipeds and other marine mammals alike, with the vast colonies of pinnipeds and seabirds of Antarctica and its unique biodiversity now under grave threat.

As Parums (2023); Puryear et al. (2023) and other authorities universally emphasize, the high virus diversity and ongoing virus reassortment events of HPAI globally, together with reports of sporadic transmission events to humans, increasingly add to uncertainty as to the potential pandemic threat to human populations of novel HPAI strains.

In 2022, World Health Organization Director-General, Tedros Adhanom Ghebreyesus, stated that the situation “needs to be monitored closely” but that the risk to humans remained low, the virus probably requiring more than one or two changes for human-to-human transmission to be a real risk, but that the impacts could be huge. Since then, in 2023, the WHO has substantially updated the tone, depth and scope of its human risk assessment of HPAI in a new significant guidance document: “With the information available so far, the virus does not appear to be able to transmit from one person to another easily, but vigilance is needed to identify any evolution in the virus that can change that.” “WHO is working closely with FAO and WOAH, and laboratory networks to monitor the evolution of these viruses, looking for signals of any change that could be more dangerous to humans. We encourage all countries to increase their ability to monitor these viruses and to detect any human cases. This is especially important as the virus is now affecting countries with limited prior experience in avian flu surveillance.” (https://www.who.int/news/item/12-07-2023-ongoing-avian-influenza-outbreaks-in-animals-pose-risk-to-humans) Here it should be noted that the WHO 15 September 2023 High Pathogenicity Avian Influenza (HPAI) - Situation Report states that around 870 cases of human infection by H5N1 has been reported of which half were fatal, around 1,599 cases of H7N9 human infections from which some 600 died; around 80 cases of H5N6 from which 30 died, and 80 cases of H9N2 from which 2 died. See also Pabbaraju et al. (2014), Pohlmann et al. (2021), Webster et al. (1981b).

The successful cross-species transmission of avian influenza viruses from their natural wild bird reservoirs to humans and the establishment of adapted variants in the human population requires the crossing of several barriers. Understanding the changes that an animal influenza virus must undergo to cross these barriers and adapt to a human host to eventually become a pandemic influenza virus is essential for better pandemic preparedness. These barriers can be divided along three major steps defining cross species transmission: (1) animal-to-human transmission barriers; (2) virus–cell interaction barriers; and (3) human-to-human transmission barriers. The nature of these barriers as well as the strategies and ability of influenza viruses to cross them are the subject of review by Reperant et al. (2012) to which more recent research is increasingly adding important data [see reviews of Charostad et al. (2023) and Szablewski et al. (2023)]. See also Lai et al. (2016).

Of particular significance in regard to spillovers of HPAIs to humans is a study by the MRC-University of Glasgow Centre for Virus Research which has found that there is a gene – the BTN3A3 gene – in humans which prevents most avian influenza viruses moving from birds to people. The gene is present in all humans and is found in the lungs and upper respiratory tract, where influenza viruses replicate. This gene was already known, but its antiviral abilities are a new discovery. However, the study found that some avian and swine influenza viruses have genetic mutations that allows them to pass the blocking effect of the BTN3A3 gene and infect people, the researchers concluding that all human influenza pandemics, including the 1918 Spanish flu and the 2009 swine flu pandemics, were a result of BTN3A3-resistant strains. R.M. Pinto, the study’s lead author, in an interview with the Guardian in June 2023, stated that the discovery was expected to have immediate practical applications. “Now, when we find cases of bird flu, we can basically swab sick birds, carcasses or feces and find out whether the virus can overcome the BTN3A3 gene, simply by looking at its sequence and determining if this virus is more or less likely to jump into humans. If the virus can in fact overcome BTN3A3 then stricter measurements should be put in place to prevent spillovers.” (Kevany, 2023; Pinto et al., 2023).

Although assessments by the WHO, OFFLU, CDC, EFSA, ECDC, FAO and WOAH and other regional and international health organizations (listed by World Health Organisation (WHO) (1967), World Health Organisation (WHO) (2002), World Health Organisation (WHO) (2022), World Health Organisation (WHO) (2023a, b, c, d), World Organisation for Animal Health (WOAH) (2022); World Organisation for Animal Health (WOAH), 2023; World Organisation for Animal Health & FAO (2023), Vallat et al., 2006; OFFLU Report, 2022; OFFLU, 2023) advise that increasing AIV infections in both farmed and wild animal species present a low known risk to humans, it is nevertheless considered imperative that the emergence of novel highly pathogenic strains subtypes should be continually monitored for the possible transmissibility from non-human species, particularly through genetic re-assortment between avian, mammal and human influenza A viruses. Here it should be noted that human infections have occurred with different subtypes of low pathogenic and highly pathogenic avian influenza A viruses. The designation of “low” versus “highly” pathogenic avian influenza A virus refers to specific criteria, including mortality in experimentally infected poultry, and not to the severity of illness with human infections. Clinical illness associated with human infections with avian influenza A viruses does not necessarily correlate with virus pathogenicity in infected birds (https://www.cdc.gov/flu/avianflu/reported-human-infections.htm)Although primarily a natural process, the emergence of highly pathogenic viral reassortants, in particular the rapidly spreading H5N1 subtype, has likely been accelerated by anthropogenic transformational stresses on the natural environment, including those causing climate change and environmental pollution. In some cases, a virulent virus such as H5N1 could thus conceivably be a key factor leading to the extinction or near extinction of certain species, such as the Californian condor (U.S. Fish and Wildlife Service California Condor HPAI Response Update, (2023)) or African penguins in southern Africa, which are already endangered or vulnerable due to other causes (Abolnik et al., 2023; https://nos.nl/artikel/2505758-vissers-en-afrikaanse-pinguins-ruzien-om-hetzelfde-sardientje).

In this context, the vulnerability and rapid decline in the Caspian seal population is reflected in an internal census by the Astrakhan State Nature Biosphere Reserve of the numbers of Caspian seals counted on the Maliy Zhemchuzhniy Island and adjacent waters in the northern Caspian Sea from May 1971 to October 2020. Recent annual records for the first half of May (the time before most seals leave for other parts of the Caspian Sea) document 5,200 seals in 2016, 1,386 seals in 2017, 2,500 for 2019 and 1,138 in 2020. This clearly indicates the extreme vulnerability of the Caspian seal population to such a scourge as HPAI H5N1.

According to the monitoring of the population of the Caspian seal and development of effective means for its conservation conducted by the Institute of Ecology and Sustainable Development (Dagestan State University) and the Research Institute of Virology, Siberian Branch, Russian Academy of Sciences, observations collected from some 100 fishermen from fishing communities from Sulak northwards along the coast of Dagestan, there were no seals to be seen on Maliy Zhemchuzhniy Island in the spring of 2023 and extremely reduced numbers were observed in adjacent waters during their seasonal migration to the south. Offshore, fewer gulls than usual were also noted by fisherman in their habitual fishing grounds. This clearly indicates ecological stress in this region, of which the reduced numbers of both seals and key aquatic birds are symptoms. Reduced ice coverage in the northernmost sector of the Caspian Sea has been considered as contributing factor to seal population decline, as this restricts the on-ice breeding zone of the Caspian seal. However, aerial surveying of this zone revealed that ice coverage of both the Russian and Kazakh part of this zone was extensive in the winter of 2022-2023 and it will be of interest to judge what effect this may have on population numbers. (http://kaspika.org/en/2023/03/01/caspian-seals-aerial-survey-in-russian-waters-2/).

With increasing momentum since 2005 when H5N1 began to have a catastrophic impact on both farmed and wild birds in Europe (Lee et al., 2017), in response to inter-related global concerns about public and animal health, as well agriculture income viability and food security in poorer regions, a number of inter-regional and international organizations have implemented transboundary surveillance and early warning processes for HPAI viruses and other infectious animal pathogens, particularly in the northern hemisphere. While some, such as WOAH and UFFLU, can be classed as primarily as data banks in that they receive and then globally disseminate relevant information provided by national health entities, in others monitoring activities are directly targeted at systematic sampling in the field, followed by virus identification and analysis by state veterinary authorities or specialized research centers, which are then followed by notifications at the national level and onwards to WOAH. In the latter category are both one-time surveillance programs implemented during sporadic (often annual) periods (e.g. Global Avian Influenza Network for Surveillance of Wild Birds) or programs which are of active, ongoing duration. See also: Wilcox and Aguirre (2004), DeLiberto (2009), Halliday et al. (2007), Hoye et al. (2010), DeLiberto et al. (2012), Machalaba et al. (2015); Short et al. (2015); Centers for Disease Control and Prevention (CDC), 2022 and Centers for Disease Control and Prevention (CDC), 2023a; Food and Agriculture Organization of the United Nations (FAO).

Notable national and regional AIV surveillance programs are in place in China (CASCIRE – see below), Japan, Kazakhstan, the European Union (European Food Safety Authority, European Centre for Disease Prevention and Control and European Union Reference Laboratory for Avian Influenza), North America (USA Departments of Agriculture and Interior with the Canadian Interagency Wild Bird Influenza Survey and Mexican government agencies), the Caribbean, Peru and Chile (Parmley et al., 2008). See Duan et al. (2023) for a current overview of avian influenza strategies and modes and the EFSA’s proposal for an active EU–level surveillance program (Walderström et al., 2022).

**Figure 13:**
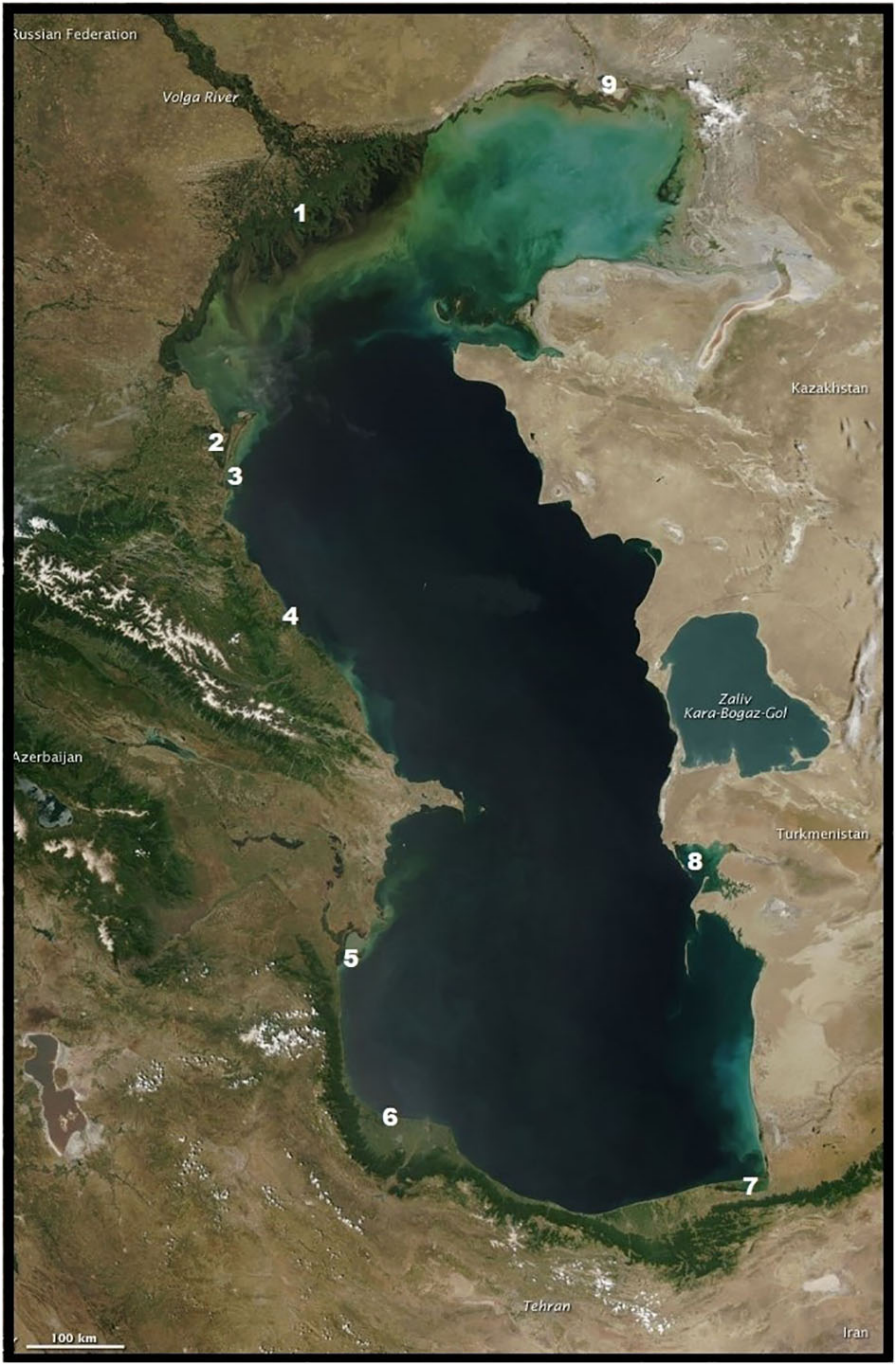
Locations of migratory aquatic avifauna stop-over sites proposed along the Caspian Sea littoral for the ViEW (Virus Early Warning) transboundary surveillance program for AIV. After: Petherbridge et al. (2022).

A critical gap in the northern hemisphere biogeographical surveillance process is the eco-corridor linking Central Eurasia/western Siberia through the Ob and Ural River and Caspian Sea basins to SW Asia and further south to the African continent. It was to bridge this global surveillance gap, that the Viral Early Warning System (ViEW) for this region was proposed in 2022 (Petherbridge et al., 2022) (**Figure 13**).

It builds on the foundation of periodic surveillance by regional scientists and responsible health authorities, notably through the collaboration of the Research Institute of Virology, Siberian Branch, Russian Academy of Sciences (RIV FRCTFM SB RAS) and the Institute of Ecology and Sustainable Development of Dagestan State University (IESD DSU) (Gulyaeva et al., 2018c; Alekseev et al., 2019).

**Figure 14:**
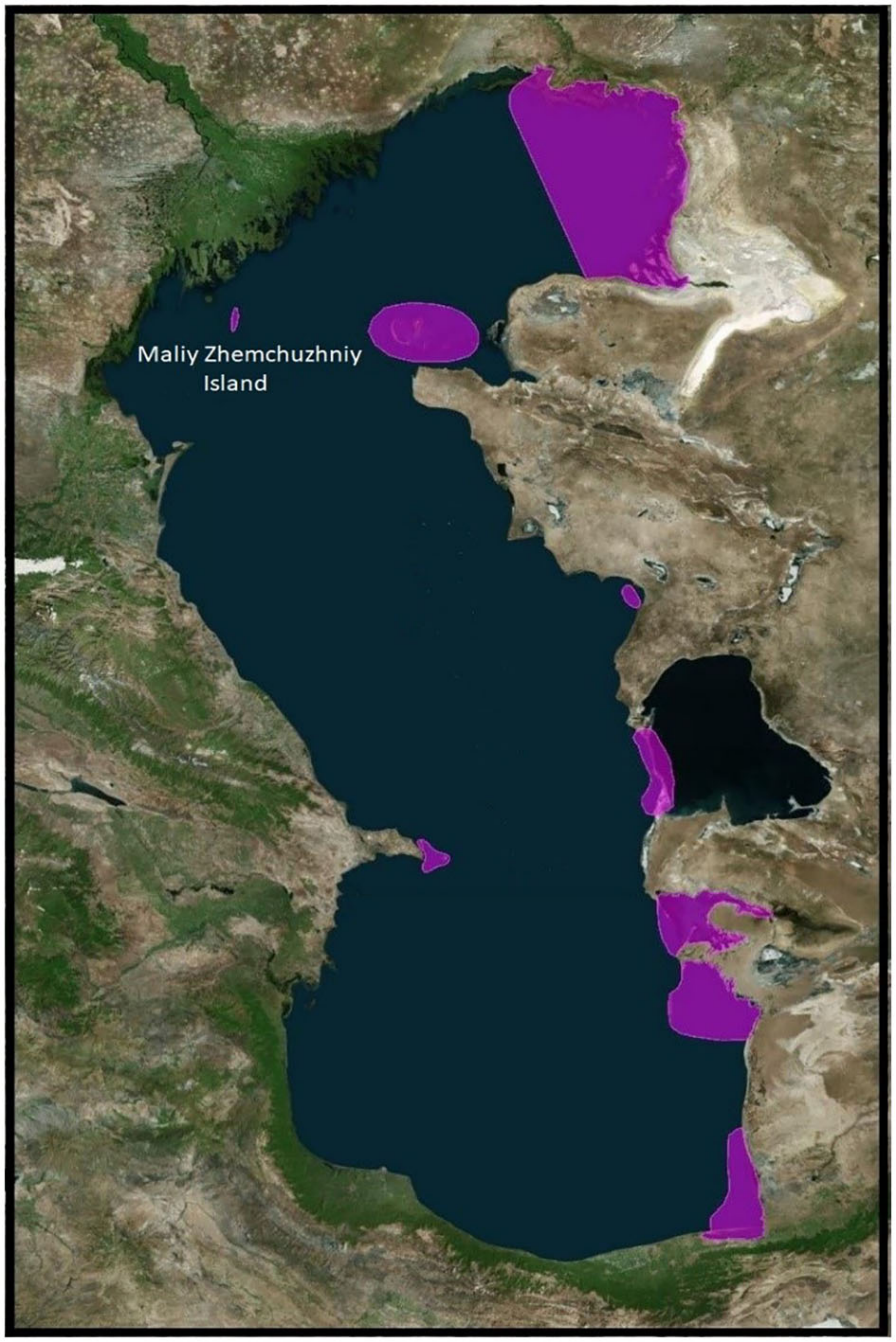
Locations of principal Caspian seal molting and haul out habitat zones in the Caspian Sea as mapped by the IUCN Marine Mammals Protected Areas (IMMA) Task Force in 2016. These are also seasonal stop-over sites for migratory aquatic avifauna. They are proposed for the ViEW (Virus Early Warning) transboundary surveillance program for AIV in seals.

The current updated proposal for ViEW, in response to the present review, is that it would integrate both Caspian seal and avifauna monitoring (Petherbridge et al., 2023) (**Figure 14**). It is also proposed that programs integrating the monitoring of both seals and coastal birds should be put in place within the 2003 Framework Convention for the Protection of the Marine Environment of the Caspian Sea (“Tehran Convention”) and with the involvement of experienced locally-based specialists (particularly those of member institutions of the Association of Universities and Scientific Research Centers of Caspian Region States) who understand not only the scientific issues but who are also best able to navigate the historical, political and socio-cultural contexts within which such programs may be effectively negotiated, established and managed.

The Chinese Academy of Sciences (CAS) Centre for Influenza Research and Early-warning network (CASCIRE) provides a comprehensive, integrated, holistic model for such a regional AIV surveillance program. It is engaged in the surveillance, genetic evolution, pathogenesis, cross-species infection identification, antivirals, antibodies and vaccine development against influenza viruses, as well as other emerging pathogens. It has the capacity to provide early-warning for outbreaks, thus leading to the development of antiviral measures (drugs, vaccines and human antibodies) for influenza viruses and other novel pathogens alongside the elucidation of pathogenesis mechanisms. Since 2014, CASCIRE and its Network Surveillance Unit have been developing a coordinated emergency response and research capacity on emerging or re-emerging infectious diseases in collaboration with partners in other states, in particular the Research Institute of Virology, Siberian Branch, Russian Academy of Sciences in Novosibirsk (Prosser et al., 2011; Bi et al., 2017; Sims et al., 2023).

The CASCIRE surveillance network monitors wildlife (e.g. wild birds), domestic and farmed animals (e.g. poultry), and includes sentinel hospitals for human cases, thus forming a complete circle for monitoring novel pathogens that pose potential risks to humans and animals alike. It is important to note that its regional monitoring process includes not only the identification of HPAI but also LPAI low pathogenicity viral pathogens, which, although they may not have a significant impact on species conservation, may provide timely early information about viral reassortants which may mutate to those of high pathogenicity to avifauna, mammals and humans. In this regard there is a need for a globally-accepted inter-regional protocol of finer granularity relating to categories of data gathered and analyses, beyond the information assembled by WOAH which reports and warns of animal diseases of high pathogenicity. Low pathogenicity H5 and H7 strains occur widely in poultry and aquatic wild birds and LPAIs can act as “incubators” for novel AIVs, although the intercontinental spread of HPAI has received greater attention in recent years. There is the risk of an H5 or H7 virus of low pathogenicity (H5/H7 low pathogenicity avian influenza [LPAI]) becoming highly pathogenic by mutation. Some avian influenza virus strains have caused sporadic zoonotic infections principally of H5, H7 and H9 subtypes and these three subtypes have been highlighted as potential pandemic risks should additional mutations occur that support sustained human-to-human transmission (Bi et al., 2017, Cox et al., 2017).

Just as it is important that LPAIs be included in monitoring regimes, recent human pandemic history has shown that novel viral strains can quickly become dominant over others considered to be of current threat. This appears to be happening in relation to the H5N1 strain, Bi et al. in 2016 reporting that H5N6 had already gradually replaced H5N1 as a dominant subtype in poultry, especially in Southern China (Bi et al., 2016).

Wu et al. (2018) state that subtype avian influenza viruses in particular are considered as likely candidates to cause a new influenza pandemic in humans. H9N2 AIVs act as a universal recipient for novel HA and NA genes of AIV. H9N2-subtype AIVs have provided internal genes to H7N9 and H10N8 viruses which have emerged in humans in China since 2013. The H9N2 subtype is widespread in poultry throughout Asia, and the H9N2 subtype from poultry in Asia has human virus-like receptor specificity (Cui et al., 2014; Bi et al., 2016; Song and Qin, 2020) Novel AIVs, such as H7N9 and H5N6, are now spreading and undergoing dynamic reassortment with low pathogenicity avian influenza viruses (LPAIVs) (e.g. H9N2) in live poultry markets (Bi et al., 2016; Cui et al., 2014). See also Shi et al. (2023).

In China H9N2 is now the dominant subtype in both farmed chickens and ducks. H5N6 has caused severe outbreaks amongst poultry in Southeast Asia and has also been found in wild birds in some regions of Asia and even in Europe after 2014 (Bi et al., 2016). There is a real risk that H5N6 may also transmit globally, following the footsteps of H5N1 and H5N8 (Global Consortium for H5N8 and Related Influenza Viruses, 2016). The potential for possible future impacts on mammals, such as pinnipeds, of virulent novel Hx reassortants can only be assessed and forecast through interlinked inter-regional networks globally with effective early warning mechanisms against emerging (and re-emerging) infectious diseases (Bi et al., 2017; WOAH, 2023).

In conclusion, the compilation of findings assembled here and the appended bibliography provide an informational toolkit for those engaged in the conservation of the Caspian seal and Caspian Sea regional biodiversity to understand the threats posed by HPAIVs and to develop and apply appropriate research and conservation management strategies. The imperative to devise, negotiate and implement a dependable, regular transboundary avian influenza surveillance program which monitors the interface between both seals and wild birds in the five Caspian Sea littoral states, as well as AIV in wildlife in adjacent regions, is clear: “To foresee is to be forewarned.”

## Author contributions

AG: Project administration, Writing – original draft, Formal analysis, Writing – review & editing, Methodology, Data curation, Conceptualization. GP: Visualization, Investigation, Writing – review & editing, Data curation. KS: Visualization, Writing – review & editing, Investigation, Data curation. IS: Writing – review & editing, Investigation, Data curation. AA: Visualization, Writing – review & editing, Investigation, Data curation. MG: Writing – review & editing, Investigation, Data curation. KL: Visualization, Methodology, Writing – review & editing. IB: Supervision, Resources, Investigation, Formal Analysis, Data curation, Methodology, Writing – review & editing. AT: Writing – review & editing. AZ: Writing – review & editing. MD: Writing – review & editing. AS: Writing – review & editing.

## Supplementary material

Supplementary File 1 The authors, originating and submitting laboratories of the sequences from GISAID’s EpiFlu™ Database on which this research is based.
